# Autochthonous Austrian Varieties of *Prunus avium* L. Represent a Regional Gene Pool, Assessed Using SSR and AFLP Markers

**DOI:** 10.3390/genes12030322

**Published:** 2021-02-24

**Authors:** Elisabeth Schüller, Felicidad Fernández Fernández, Laima Antanaviciute, Ulrike Anhalt-Brüderl, Andreas Spornberger, Astrid Forneck

**Affiliations:** 1 Division of Viticulture and Pomology, University of Natural Resources and Life Sciences, Vienna Gregor-Mendel-Strasse 33, 1180 Vienna, Austria; elisabeth.schueller@boku.ac.at (E.S.); ulrike.anhalt@boku.ac.at (U.A.-B.); andreas.spornberger@boku.ac.at (A.S.); 2 Genetics, Genomics and Breeding Department, NIAB EMR, New Road, East Malling, Kent ME19 6BJ, UK; felicidad.fernandez@niab.com; 3 QIAGEN, 19300 Germantown Road, Germantown, MD 20874, USA; laima.antanaviciute@gmail.com

**Keywords:** sweet cherry, *Prunus avium*, breeding, Austrian germplasm, autochthonous varieties, genetic diversity, simple sequence repeat (SSR), amplified fragment length polymorphism (AFLP)

## Abstract

Sweet cherry production faces new challenges that necessitate the exploitation of genetic resources such as varietal collections and landraces in breeding programs. A harmonized approach to characterization is key for an optimal utilization of germplasm in breeding. This study reports the genotyping of 63 sweet cherry accessions using a harmonized set of 11 simple sequence repeat (SSR) markers optimized in two multiplexed PCR reactions. Thirty-eight distinct allelic profiles were identified. The set of SSR markers chosen proved highly informative in these germplasm; an average of 6.3 alleles per locus, a PIC value of 0.59 and above-average expected and observed heterozygosity levels were detected. Additionally, 223 amplified fragment length polymorphism (AFLP) markers derived from eight selective primer combinations were employed to further differentiate 17 closely related accessions, confirming the SSR analysis. Genetic relationships between internationally known old cultivars were revealed: SSR fingerprints of “Schneiders Späte Knorpelkirsche” and “Germersdorfer” were found to be identical to those of the standard cultivar “Noire de Meched”, among others, whereas four accessions known as “Hedelfinger Riesenkirsche” and four known as “Große Schwarze Knorpelkirsche” showed allelic differences at various loci. The genetic diversity of locally-grown cultivars worldwide might be currently underestimated. Several autochthonous Austrian sweet cherry germplasm accessions were genotyped for the first time and their genetic relationships analyzed and discussed. Interestingly, seven Austrian sweet cherry landraces were shown to be clearly genetically separated from international and modern varieties, indicating that Austrian germplasm could include valuable genetic resources for future breeding efforts.

## 1. Introduction

Autochthonous varieties and their wild relatives are valuable genetic resources for the breeding and development of any domesticated crop [1,2,3,4]. Global climate change is a key driver for some of the challenges that sweet cherry production must address, such as double fruits [5] or fruit cracking [6] stem. The availability of well characterized genetic resources from different climatic zones and growing regions will continue to gain importance, especially since r modern sweet cherry breeding programs have focused on few selected genotypes as parents causing a severe genetic bottleneck [2]. Future breeding programs aiming to support sustainable sweet cherry cultivation will need adequate and diverse germplasm to answer to these challenges. The need for a harmonized approach to genetic characterization of national and international germplasm pools to maximize their usefulness as resources for breeding and research has been highlighted in various European-wide initiatives e.g. the European Cooperative Programme for Plant Genetic Resources (ECPGR) [7] and the European Cooperation in Science and Technology (COST) action FA1104 (http://www.cost.eu/COST_Actions/fa/FA1104). 

Despite a long-standing history of sweet cherry growing [8], the economic importance of this crop in Austria has decreased drastically over the last decades: in 1995, 28.7 kt of sweet cherry were produced in extensive production systems—compared to only 4.1 kt in 2017, of which only around 1.4 kt were harvested from intensive commercial plantings in 2017 [9]. Only a handful of modern cultivars are used commercially mainly due to a strong emphasis on large fruit size by retailers. Little is known about the occurrence, identity and diversity of old and locally developed Austrian landraces still grown in the mostly abandoned traditional extensive production systems [8,10]. 

Recent surveys of sweet cherry landraces in the provinces Burgenland [11,12] and Upper Austria [13] provide detailed morphologic descriptions of 71 sweet cherry varieties, 19 of which could be identified based on pomological descriptions, whereas 52 distinct phenotypes could not be identified due to the lack of references in the literature. These were presumed to be autochthonous Austrian landraces. 

Environmental and phytosanitary factors can lead to morphological variation in plants [14], potentially calling into question the accuracy of the varietal identification on phenotypes alone [15,16,17]. This limitation is particularly severe when working with clonally propagated perennial fruit tree species. Comparing specimens of diverse age, health and unclear phytosanitary status of rare varieties, as in the case of the above-mentioned germplasm surveys, can prove extremely challenging. 

The deployment of genetic markers such as simple sequence repeats (SSRs), or single nucleotide polymorphisms (SNPs) to reveal a characteristic genetic fingerprint, considerably accelerates and simplifies the process of cultivar grouping and identification [15,18,19,20]. The analysis of the alleles at the self-incompatibility locus (S-alleles) has also been used in genetic studies in sweet cherry; it provides valuable genotypic information that is very relevant for fruit growing and production [21,22,23]. SSRs have been the marker of choice to analyze diversity and genetic structure in sweet cherry [15,16,19,24,25] over the last twenty years, and the ECPGR still recommends a standard set of 16 microsatellite markers and the inclusion of eight reference accessions in diversity studies to harmonize future fingerprints of sweet cherry collections with already existing datasets. This harmonization should enable the detection of synonyms and duplicates in and across collections as well as the verification of the phenotype-based identification of accessions. A reliable confirmation of “trueness-to-type” is key to help rationalize collections and to obtain comparable data for different sweet cherry germplasm sources [7].

SSR markers inherently cover a very limited part of the target genome. They are not universally polymorphic. A defined set of markers could reveal genetic polymorphism within one population but be less informative for a second group of genotypes, e.g., closely related landraces from another gene pool [25,26,27]. Amplified fragment length polymorphism (AFLP) markers have been identified as a powerful tool for differentiation between closely related individuals at the population level [18,28] and have been successfully used in sweet cherry [19,29,30]. A genome-wide 6K SNP array was developed in 2012 for genetic studies and breeding in sweet and sour cherry, but only a third of the SNPs were found to be informative [20]. This SNP array has only very recently been improved [31] and promises to become a powerful tool in sweet cherry research. For this study, SNP arrays were not applied. 

This is the first study reporting genetic fingerprints of autochthonous Austrian landraces and germplasm accessions of sweet cherry. The research questions we wanted to address were the following: (1) Are the SSR marker set and the multiplex method we chose effective? Do they provide fast and reliable results that can be harmonized? (2) Can Austrian sweet cherry landraces be successfully differentiated by the selected set of SSR markers? (3) Have the selected accessions and landraces been correctly identified? 

## 2. Materials and Methods

### 2.1. Plant Material

Plant material for SSR-analysis was collected at two locations. (1) Sample Set STB: Stoob, Burgenland, AT (*n* = 29) representing old landraces and probably regional selections of known cultivars in the traditional high-stem meadow orchard growing system; and (2) Sample Set BOK: University of Natural Resources and Life Sciences, Vienna (BOKU) germplasm collection, Vienna, AT (*n* = 23) including Austrian landraces and some modern varieties. Additionally, ECPGR reference genotypes from East Malling Research, United Kingdom (GB) (*n* = 5) and United States of America (US) reference genotypes (*n* = 6) were included in Sample Set IS. These reference genotypes were used to standardize the SSR data as described by [7]. The individual samples were given a code (A01–A63) and named according to their phenotype-based identification of a known variety or cultivar, respectively, or using the accession name in the germplasm collection. Varieties that could not be reliably identified were referred to by their tree numbers (TN). In this study, the term “accession” is used to address the individual plant in the collection or sample set, respectively. Tree numbers identify the individual tree in the collection (Table 1).

AFLP analysis was conducted to further investigate the genetic identity and relationships of certain accessions showing identical SSR fingerprints. With AFLP analysis it is possible to detect polymorphisms between samples, covering the whole genome without prior knowledge of the DNA sequence [32]. Therefore, it is considered a suitable technique to differentiate between very closely related individuals of the same species [18,28]. We tested a subset of the BOKU germplasm accessions against the ECPGR standards “Noire de Meched” and “Noble” as well as landraces from two Austrian sweet cherry growing regions. 

Plant material for the AFLP-analysis was collected at three locations: (1) Leithaberg, Burgenland (*n* = 4) (2) Scharten, Upper-Austria (*n* = 5) and (3) BOKU germplasm collection *(n* = 8), Vienna. ECPGR reference genotypes “Noire de Meched” and “Noble” were included in the analysis (Table 2). Samples from Stoob were not included in this analysis. For simplification of the graphs, samples are coded by their TN instead of variety names.

### 2.2. DNA Extraction

For SSR analysis, genomic DNA was extracted from fresh leaves or winter buds with the DNeasy Plant Mini Kit (QIAGEN, Chatsworth, CA, USA) and diluted to a concentration of 2.5–4 ng/µL. For the reference accessions, lyophilized leaf-samples were extracted and diluted accordingly. The genomic DNA for the US reference accessions was kindly provided by Dr Amy Iezzoni’s team in Michigan State University.

For AFLP analysis, DNA was extracted from lyophilized leaf-samples according to [33] with minor modifications.

### 2.3. SSR Analysis

Twelve primer pairs recommended by the ECPGR [7] and suitable for multiplexing were combined in two multiplex (MP) PCRs depending on the fragment sizes of amplified PCR products: MP1: large (173–261 bp) and small (98–187 bp), MP2: medium (120–208 bp). Primers were labeled with fluorescent dyes (Table 3).

Primer concentrations in PCR reactions were optimized to produce similar intensity of chromatogram peaks to facilitate scoring. The final volume of PCR reactions was 13 µL containing 5–8 ng genomic DNA, 2× Type-it Multiplex PCR Master Mix (QIAGEN, Hilden, Germany), 1.25–5 nmol of primer, the exact quantity depending on each primer (Table 1).

PCR was started with a denaturing step at 95 °C for 5 min, followed by 10 circles of touchdown-PCR: 95 °C for 30 s, 60 °C for 1.5 min (−1 °C per cycle) and 72 °C for 30 s, followed by 18 cycles: 95 °C for 30 s, 50 °C for 1.5 min and 72 °C for 30 s, with a final elongation step of 60 °C for 30 min.

The amplicon sizes were measured against a LIZ 500 standard with an ABI 3130 Genetic Analyzer (Applied Biosystems, Waltham, MA, USA). Subsequent scoring was done with GENESCAN^®^ and GENOTYPER^®^ (Applied Biosystems, Waltham, MA, USA). Scored fragment sizes were harmonized with those of the reference genotypes using the allele sizes published by [7]. 

### 2.4. AFLP-Analysis

AFLP analysis was performed following the protocol by Vos et al. with the following modifications: Genomic DNA (0.3 µg) was incubated with 3.6 U Tru1I and 45 U EcoRI in 25 µL Tango-Buffer (Fermentas, St. Leon-Rot, Germany) for 1 hour at 37 °C, followed by two hours at 65 °C and 15 min at 85 °C. 5 µL of the adapter-ligation-solution as described by Vos et al. [40] were added and incubated overnight at 20 °C followed by a 1min-step at 65 °C to inactivate the T4 Ligase. For preamplification the template was diluted 1:5 and 2.25 µL were incubated in a 15 µL-PCR reaction with Taq DNA polymerase (5 U/µL) and 10× Taq buffer (Fermentas, St. Leon-Rot, Germany), 1.5 pM of each AFLP-primer without selective extensions, 3 mM MgCl_2_, 0.2 mM of dNTPs. The pre-amplified template was diluted 1:10 and 4 µL were incubated in a 10µL-PCR reaction with 0.5 pM of each of the selective primers. For this final selective amplification, 18 different primer combinations were tested on nine varieties. The eight most promising combinations were selected for the analysis: ATC/ATC, ACC/ATC, ACC/CAG, AGG/AGT, AGG/CAG, ATA/ATC, ATA/AGT, ATA/CAG. AFLP-fragments were run on a LI-COR (NEN Model 4300) analyzer (LI-COR Inc., Lincoln, NE, USA) and scored for presence (1) or absence (0) by hand with SagaTM (Version 3.0) (LI-COR inc., Lincoln, Nebraska USA). For all samples, except two, biological replicates (separate leaves, DNA extractions, digest, ligation, preamplification and selective amplification) for estimation of clonal variation were done and run side by side. Additionally, a technical control was run for four samples (same DNA extraction, separate digest, etc.). Three types of negative controls, one from the start and one for each of the PCRs were included. Only clearly visible bands between 90 and 400 bp of length were scored.

### 2.5. Data Analysis and Statistics:

For SSR data and allele frequencies, the identification of unique alleles was done in GenAlEx version 6.5 [41,42]. polymorphism information content (PIC)-values were subsequently calculated in Excel according to 43 [43].

**Figure 1:**
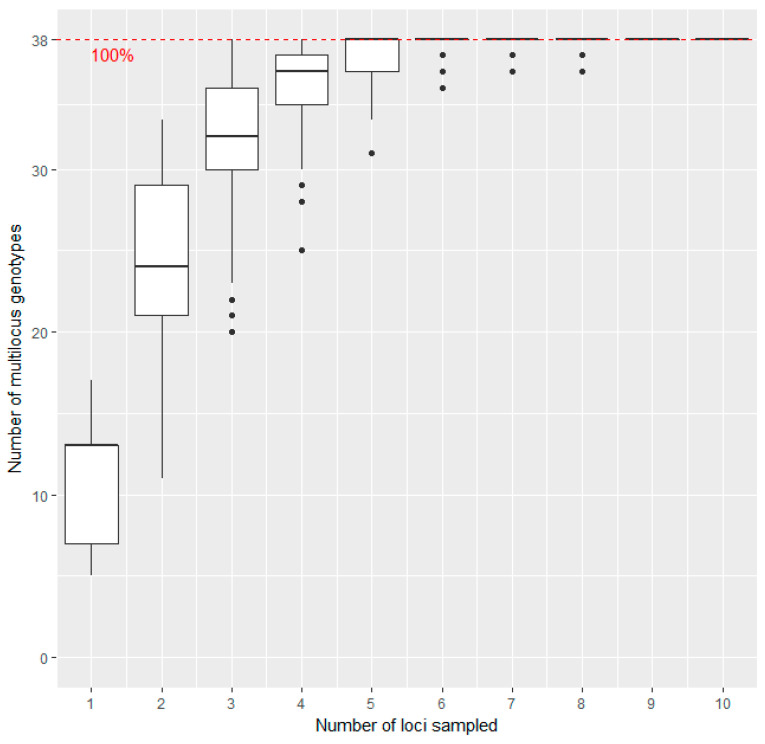
Genotype association curve for SSR analysis of sweet cherry accessions: The horizontal axis represents the number of SSR loci randomly sampled from the dataset, while the vertical axis shows the number of Multilocus Genotypes (MLGs) observed. The red dashed line represents 100% of the total MLGs observed in the data set. The plateau is reached with five loci sampled, and variance decreases to a minimum with six loci.

The calculation of the genotype association curve (Figure 1), frequency based diversity estimators, allelic richness, observed heterozygosity, expected heterozygosity, the distance matrix, the dendrogram with Nei’s distance as well as the genetic diversity indices for the clusters resulting from Discriminant Analysis of Principal Components (DAPC) were done in the statistics environment R version 4.0.3 [44] using packages poppr version 2.3.0 [45,46] and adegenet version 2.1.3 [47,48].

The genotype association curve (Figure 1) reveals that with five loci sampled already 100% of the Multilocus Genotypes (MLGs) across the sampling set tested could be detected. We conclude that the method using 11 SSR markers has adequate power to discriminate between the unique individuals in our dataset and adding more markers would not reveal many additional genotypes.

The DAPC was done in R, package adegenet version 2.1.3 [47] for SSR -data. In contrast to the Principal Component Analysis (PCA) or Principal Coordinate Analysis (PCoA), this analysis doesn’t focus on the global genetic variation resp. diversity of the dataset, but instead optimizes the discriminant functions which show differences between groups, minimizing variation within clusters [49]. 

For AFLP-scores the PCoA was done in GenAlEx [41,42]. This analysis displays the overall genetic diversity present in the dataset.

## 3. Results

### 3.1. Method Evaluation of SSR-Analysis

Eleven of twelve SSR markers gave unambiguous results in the multiplex approach, which could be standardized. Scores of BPPCT034 [39] could not be standardized, because the allele lengths of the reference accessions were found to be ambiguous. Data for this marker were excluded prior to analysis. The remaining 11 markers were amplified in two multiplexed reactions, resulting in a fast and easy-to-use fingerprinting system. The calculated genotype association curve (Figure 1) showed that the number of loci obtained with these markers was sufficient to cover the genetic diversity present in the sample set. Other studies used similar numbers of makers for genetic fingerprints in *Prunus avium*, obtaining reliable results [7,19].

The number of alleles per locus ranged from 3 to 9, with an average of 6.3; PIC values ranged between 0.22 (EMPa017) and 0.78 (BPPCT037), with an average of 0.59. The observed and expected heterozygosity ranged between 0.06 resp. 0.23 (EMPA017) and 0.89 resp. 0.81 (BPPCT037) depending on the SSR marker (Table 4). 

Eight different genotypes (four genotypes from Stoob, two from the BOKU collection and two international standards) show unique alleles in up to three markers. The best markers in terms of detecting unique alleles in different genotypes were CPPCT022 and EMPaS10, with unique alleles amplified in six and five genotypes, respectively.

### 3.2. Variety Identification and Verification of Trueness to Type

The genotypes obtained by SSR fingerprinting allowed us to confirm or reject the morphological identification of landraces and germplasm accessions and revealed the occurrence of homonyms and synonyms in otherwise well-known varieties. Some phenotyping, grafting or labelling errors were also detected (e.g. “Burlat VG” which is not identical with the cultivar “Bigarreau Burlat”; “Lambert” and “Stella Spur” which surprisingly showed the same fingerprint). Four trees in Stoob were phenotyped as cultivar “Große Schwarze Knorpelkirsche”; only two showed the same genotype and all of them differed compared to the reference accession in the BOKU germplasm collection. Two of three trees phenotyped as “Hedelfinger Riesenkirsche” and collected in Stoob had the same fingerprint, but all of them differed from the two accessions of the same name in the BOKU germplasm collection, which also turned out to show two distinct genetic profiles. Some clonal variants were also identified such as the samples phenotyped as landrace “Butterkirsche” and the two types of “Kritzendorfer Einsiedekirsche”. Six of the sampled Austrian landraces showed to be identical to several germplasm accessions and standard cultivars from different origins. This group of seemingly identical genotypes includes the accessions “Germersdorfer”, “Schneiders Späte Knorpelkirsche”, the ECPGR reference cultivar “Noire de Meched” and the US-reference “Schneiders”, as well as traditional Austrian cultivars like “Melker Riesenkirsche” and “Horitschoner Herzkirsche”. For simplification purposes this group of accessions will be mentioned as the “Schneiders-Group” throughout this study.

An AFLP-analysis including further samples from two other sampling sites was conducted to confirm if these cultivars are true clones i.e., synonyms (see below). 

### 3.3. Genetic Diversity

**Figure 2:**
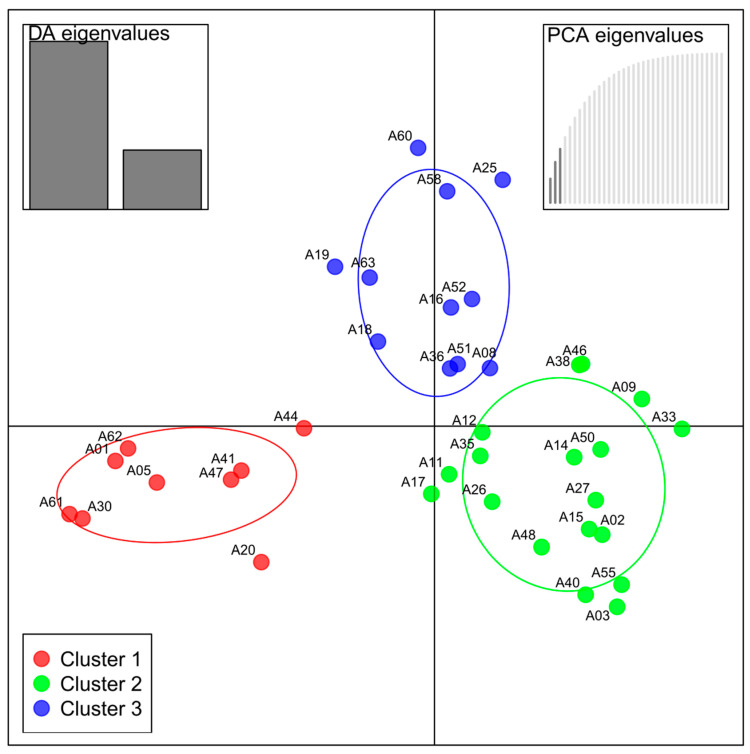
Scatter plot of discriminant analysis of principal components (DAPC) for SSR-data, three PCs and three DAs retained. Color code and group-ellipses according to the three calculated clusters. The box on the upper left shows the retained DA eigenvalues, while the box on the upper right shows that three PCA eigenvalues have been retained.

**Figure 3:**
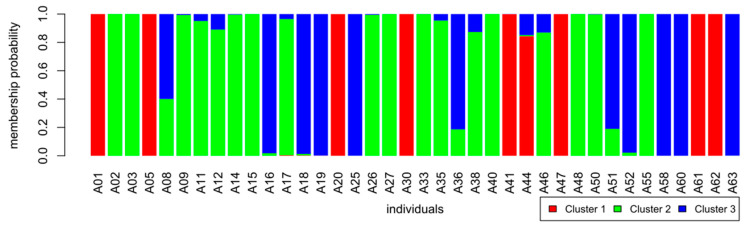
Membership probability of samples to the three identified clusters. Depiction of admixture. X-axis: individual samples, y-axis: membership probability to one of three clusters: 1: red, 2: green, 3: blue.

The Discriminant Analysis of Principal Components (DAPC, R, adegenet) was used to identify groups of genetically related individuals in all three sample sets. This analysis has been developed and is suitable for clonal or partly clonal populations, since it does not rely on Hardy–Weinberg or linkage disequilibria [50]. The varieties group into three defined separate clusters (Figure 2); each cluster contains members of the three sample sets: BOK = BOKU germplasm collection, IS = International Standard, STB = Stoob. Part of the samples showed to be admixed according to the estimated probability of group membership, which is depicted in the membership probability plot (Figure 3).

Cluster 1 consists of nine multi-locus-genotypes (MLGs) and contains landraces from Burgenland (“Butterkirsche”, “Sämling von Sauerbrunn” and “Donnerskircher Blaukirsche”) as well as the rootstocks “NY54” and “F12/1” (Table 1 and Table 5). Only one of these samples (“Kritzendorfer Einsiedekirsche”) shows to be admixed with cluster 3. The other two clusters share more admixed genotypes (Figure 3). In cluster 2, 18 samples or MLGs are found; some of the modern cultivars, all samples of the two widely distributed cultivars “Große Schwarze Knorpelkirsche” and “Hedelfinger Riesenkirsche” and some landraces denominated by tree numbers (TN). Cluster 3 comprises of the rest of the modern cultivars and the “Schneiders-Group” which was included in the analyses represented by one sample of this genotype: TN39 (Table 5).

According to Simpson’s diversity index (lambda, Table 6), DAPC-cluster 2, which includes many accessions of the germplasm-collection, is the most diverse, with 0.944, followed by cluster 3, with 0.90. Cluster 1 is the least diverse, with lambda 0.889; this cluster mostly consists of the landraces from Burgenland, which, unsurprisingly, were shown to be very closely related to each other.

The expected heterozygosity (Hexp) of all three clusters were very similar, with values ranging from 0.595 (cluster 2) to 0.63 (cluster 1).

**Figure 4:**
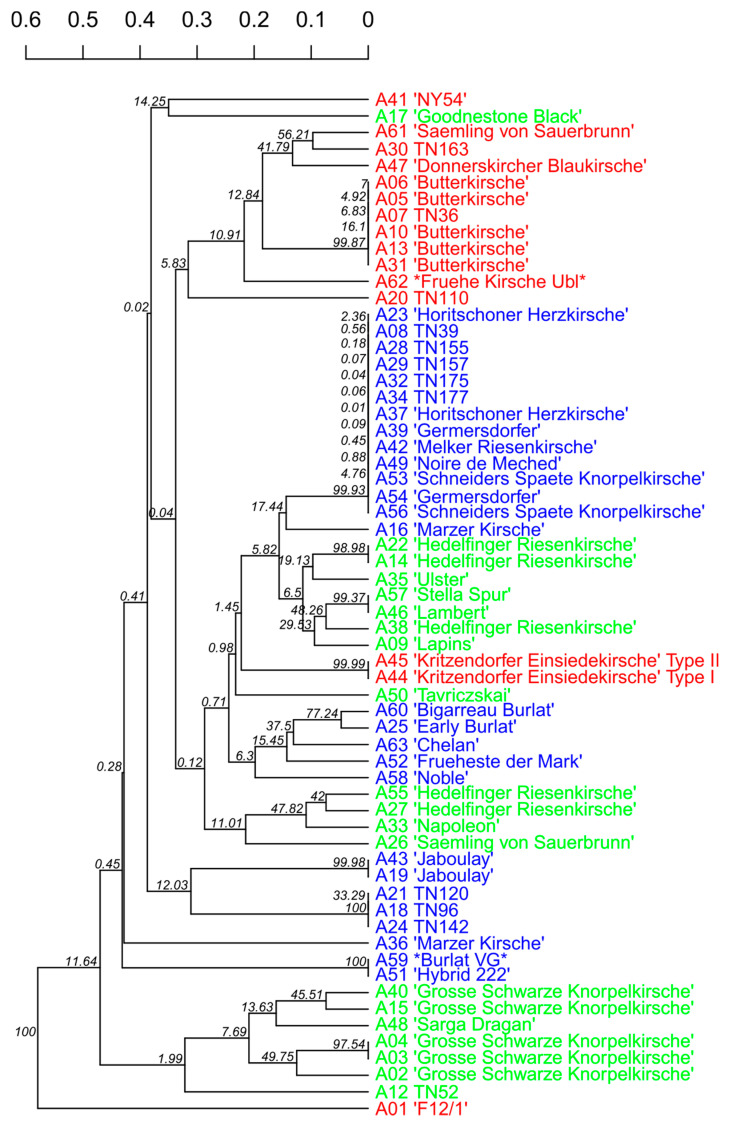
Dendrogram based on SSR data, Nei’s distance, bootstrapping (10,000), unweighted pair group with arithmetic means (UPGMA); color code according to DAPC clusters: 1: red, 2: green, 3: blue.

The dendrogram (Figure 4) was calculated based on Nei’s distance, bootstrapping (10,000) and UPGMA (unweighted pair group with arithmetic means). Variety A11 TN46 had to be excluded for this calculation, because the algorithm based on Nei’s distance cannot process missing data, and the SSR marker CPSCT038 did not amplify any PCR products for this genotype (see also Supplement-File S1). The dendrogram shows four main clusters of sweet cherry varieties. Though based on the same data, the clusters are composed differently compared to the DAPC (Figure 2, Table 3), as they are calculated using a different algorithm. 

DAPC-cluster 1 (red) is represented on the upper end missing “Kritzenorfer Einsiedekirsche”, which groups with the varieties of the cluster below. NY45 groups together with Goodnestone Black (uppermost end), and F12/1 appears separated from all other varieties on the bottom end of the dendrogram. The other two DAPC clusters appear to be admixed, whereas notably all genotypes of “Hedelfinger Riesenkirsche” appear in one cluster and all genotypes of “Große Schwarze Knorpelkirsche” in another. A separate cluster consists of “Hybrid 222”, i.e., “Burlat VG”. Unlike DAPC analysis, the dendrogram shows the rootstock “F12/1” as more genetically distant from the rest of the varieties (Figure 4). This might be a result of the clustering algorithm UPGMA assuming the occurrence of a hierarchical structure between the individuals and rooting this structure in one sample, which might not be an accurate assumption for our data set. The low bootstrap-values on the left-side nodes indicate that the separation of the main clusters as shown in the dendrogram is not very well supported by the data. This is probably due to the limited amount of processed data, combined with the fact that the sweet cherry varieties in this study are generally very closely related.

### 3.4. Results from AFLP-Analysis

For AFLP-analysis samples of “Germersdorfer” (*n* = 7), “Horitschoner Herzkirsche” (*n* = 3), “Melker Riesenkirsche” (*n* = 2), “Schneiders Späte Knorpelkirsche” (*n* = 3) of three origins and the ECPGR standard “Noire de Meched” were tested. We also included the clearly distinct phenotypes “Noble” (which also shows a different SSR-fingerprint), rootstock “F12/1” as well as the Austrian landrace “Rainkirsche” (K02) (Table 2). Four runs had to be excluded due to a high proportion of failed bands.

An AFLP analysis with eight selective primer combinations and 17 samples of four different origins resulted in 223 markers, of which 64 (28.7%) were polymorphic. 

**Figure 5:**
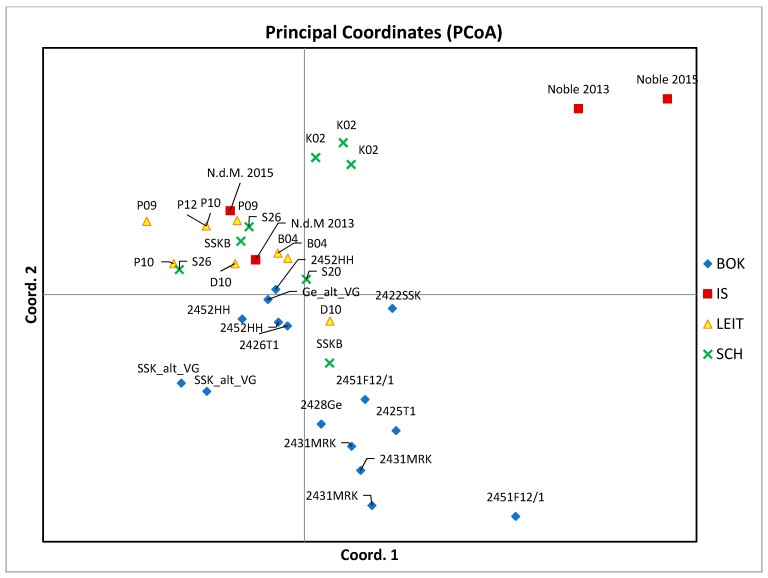
Principal Coordinates 1 and 2 (% of var. explained: 32.7%) of AFLP-data of sweet cherry accessions. Blue: BOK = BOKU, red: IS = International Standard, yellow: LEIT = Leithaberg, green: SCH = Scharten.

The error-rate was moderate, with 1.36–1.79% of difference in band-occurrence between technical replicates. Biological replicates differed up to 6.73%. The samples of “Noble” could clearly be separated from the other samples with PCoA (Figure 5). The percentage of variance explained by the first two axes was 32.7% and 44.17% by the three main coordinates. This low power of explanation of variance is probably due to the low number of samples and the generally low variation between the tested genotypes. Therefore, these results should be interpreted with caution.

## 4. Discussion

The optimized multiplex-PCR approach to SSR genotyping was broadly successful; 11 of the 12 SSR-markers were easily scored and standardized against published data for five reference accessions (data not shown). This gives us confidence in the quality of the data generated which should prove straightforward to compare with similarly standardized data for other germplasm collections in the future The number of markers was comparable to that of similar studies [3,4,23,27,51] and showed to be sufficient to reveal the genetic diversity of MLGs expected to be present in the samples analyzed. Furthermore, with an average of 6.3 alleles per locus and PIC-value of 0.59 as well as the above-average expected and observed heterozygosity-levels, it is clear that the set of SSR-markers chosen was highly informative in the sweet cherry varieties in this study. The method of characterization proved effective and provided solid results that can be reliably harmonized with future studies.

The chosen primer combination was found to be effective for differentiating between most of the Austrian sweet cherry landraces tested. Regarding the accurate identification of duplicates (i.e. synonyms) and homonyms (i.e. genetically heterogenous groups phenotyped as the same cultivar), both could be detected by SSR-analysis, providing essential information for the optimal management of the germplasm collection and future breeding approaches.

In DAPC cluster 2 (Figure 2), there are two heterogenous groups. In five samples, all phenotyped as “Große Schwarze Knorpelkirsche”, four distinct genotypes could be detected. This is understandable; this cultivar dates back to the 16th century and has been one of the most important in central Europe [52]. 

Four of five samples phenotyped as “Hedelfinger Riesenkirsche” showed differences in allele sizes. The cultivar “Hedelfinger Riesenkirsche” originates from Hedelfingen in Germany where it was selected from seedlings and taken to Hohenheim (Germany) around 1850. Thereafter, this cultivar has been widely distributed by tree nurseries [8] and is found in many places around the world nowadays often referred to as “Hedelfingen”.

“Große Schwarze Knorpelkirsche” as well as “Hedelfinger Riesenkirsche” were assigned to the same DAPC cluster; both have been referred to as “population cultivar”, indicating that the cultivar consists of several phenotypes [53]. This could be due to mixed vegetative and sexual reproduction combined with selection for a certain fruit morphology—which makes these cultivars relatively easy to phenotype—over the past decades. Another possibility is clonal variation, i.e., the vegetative propagation of sport mutants. 

On the other hand, three not previously identified genotypes of distinct phenotypes found in Stoob showed the same fingerprint (TN96, TN120, TN142; Figure 4). In that case, it has to be considered that the discriminating power of the limited set of SSR markers might not be adequate for these genotypes.

The identification of synonyms or duplicates helps to reduce the number of accessions and therefore the running costs in conservation efforts such as germplasm collections [54]. On the other hand, mutations and thus clonal variation occur, especially in long-lived tree species [55,56]. Certain clones potentially harbor desirable superior traits such as yield [27], climatic adaptation or tolerance to pests and diseases, and thus the identification of true clonal variation within germplasm collections is essential.

A group of accessions showing the same genetic SSR fingerprint includes presumably different late-ripening, heart-shaped, dark-red, firm cultivars with considerable fruit size. Surprisingly, very well-known, Europe-wide distributed cultivars like “Germersdorfer” and “Schneiders Späte Knorpelkirsche” as well as typical Austrian cultivars like “Melker Riesenkirsche” and “Horitschoner Herzkirsche” (named after Melk and Horitschon—two towns in the eastern part of Austria) are found in this group, along with the ECPGR reference cultivar “Noire de Meched” and the US-standard “Schneiders”. Although these findings could be due to limited SSR-marker resolution, suspicions that some of the cultivars could be clones and their names therefore synonyms have been raised before. Braun-Lüllemann and Bannier [52] found that in Germany the cultivar denominated as “Germersdorfer“ in the past decades is morphologically identical to the cultivar known as “Schneiders Späte Knorpelkirsche“.

This finding is especially intriguing since this latter cultivar was found to be comparable in fruit size to widely distributed modern cultivars like “Regina” or “Kordia”, and thus represents a potential candidate for breeding. Considering that maximizing fruit size still is one of the most important objectives in sweet cherry breeding [57], it is essential to find out whether the studied varieties are in fact clones. The verification of this hypothesis would probably reduce the number of suitable high-fruit-sized parent-candidates for future breeding programs. Differences in the phenotype due to clonal or epigenetic variation would have to be studied in appropriate trials, to select for the best clones. Moreover, for a cost-effective rationalization of germplasm collections, it is important to know if those Austrian cultivars are in fact all duplicates.

To our knowledge, until now there has been no such study. To gather additional evidence on the correct genetic identity of the above-mentioned varieties which have identical SSR profiles, an AFLP analysis was conducted.

We compared the ECPGR standard “Noire de Meched”, accessions of the Austrian germplasm collection “Germersdorfer”, “Schneiders Späte Knorpelkirsche”, “Melker Riesenkirsche” and “Horitschoner Herzkirsche”, as well as samples from two different sites consisting of several landraces identified as one of the just mentioned cultivars. Three genetically and phenotypically different accessions were included for comparison. 

Based on the results of the AFLP analysis, the tested varieties in general show low genetic variation; only about 29% of markers were shown to be polymorphic. This is in agreement with reported polymorphic rates of 21% for AFLP markers in sweet cherry [19]. Technical error rates are as high as 1.79%. For biological replicates (same tree, different branch), differences of up to 6.73% were recorded. These differences could be explained by clonal variation resp. sport mutation, and therefore it is also probable that morphologically identical or very similar varieties are clones of one and the same widespread cultivar. As expected, samples of the cultivar “Noble” could clearly be separated from the rest by PCoA (Figure 5). Nevertheless, samples of the other two very different phenotypes “F12/1” and “Rainkirsche” appear close to or inside the cluster of “Schneiders-Group” cultivars. This could be due to the proportion of technical errors combined with the comparatively small genetic distance between *Prunus avium* varieties, which puts more weight on such technical errors. To sum up: based on the results shown in this study genetic differences among the tested varieties exist. These genotypes might represent valuable resources for future breeding efforts, if they have superior traits, e.g., disease resistance or superior fruit size. 

The genetic diversity of the Austrian landraces evaluated was subsequently compared to that of international standard cultivars based on the results of the DAPC and calculated diversity indices. The DAPC sorted the samples into three clusters. In cluster 2, all samples of “Hedelfinger Riesenkirsche” and “Große Schwarze Knorpelkirsche” group together with “Goodnestone Black”, “Napoleon”, “Stella Spur”, “Lambert”, “Lapins”, “Ulster”, “Tavriczskai” and “Sarga Dragan”. “Chelan” groups with “Noble”, “Early Burlat”, “Burlat”, “Jaboulay” and “Früheste der Mark” in DAPC cluster 3, whereas DAPC cluster 1 comprised only varieties from Burgenland, i.e., landraces not mentioned in the available literature. These autochthonous varieties seemed clearly distinct from the other groups and may therefore constitute a valuable germplasm for breeding as members of a regional gene pool. Details on valuable phenotypic and physicochemical characteristics of these varieties such as unique taste, low susceptibility to rain-induced cracking or high content of polyphenols in the fruit have been recorded in prior studies [11,12,58]. The landraces are probably admixed with the wild cherry population of this specific region. Interestingly, rootstocks “NY45” and “F12/1” are also assigned to this cluster. 

## 5. Conclusions

A successful method of fast and easy-to-use multiplex SSR analysis for international harmonization of sweet cherry accessions was presented. The investigated collection of autochthonous Austrian sweet cherry landraces is highly diverse and could constitute a valuable germplasm for future breeding programs, since Austrian landraces were shown to represent a regional gene pool. They exhibit interesting traits that might be valuable for breeders (Table 1) and are most probably adapted to the local climate and environmental factors, since they comprise ecotypes that have been cultivated in the same region for decades. Furthermore, they might harbor certain traits like tolerance to fruit cracking or tolerances to diseases and pests, which has to be evaluated in further studies.

Concerning the various genotypes of “Große Schwarze Knorpelkirsche” and 2Hedelfinger Riesenkirsche”, marker assisted selection (MAS), field trials and cultivar evaluations should be conducted to identify the most valuable of the clones for breeding purposes.

It would be interesting to compare the genetic diversity of Austrian landraces with those from the French collection described by Mariette et al. [2]. Does it comprise a different gene pool? What is the influence of the wild cherry population in Austria and how is this gene pool different compared to the French wild cherries?

Phenotype-based surveys on sweet cherry diversity have been conducted for some Austrian regions [10,11,12,13], and Austrian landraces were shown to bear valuable characteristics such as a high content of polyphenols in the fruit [58]. While important first steps have recently been taken to preserve and protect these landraces in the future, considerable gaps of knowledge still need to be filled to effectively preserve the Austrian sweet cherry diversity.

Part of these gaps could effectively be filled by genetic evaluation, as has been shown in this study. Homonyms, synonyms and labeling errors were detected. The genetic data help to evaluate the genetic diversity, identity and trueness to type of Austrian sweet cherry accessions and thus serves as an important and valuable tool for the management of Austrian germplasm collections.

## Figures and Tables

**Table 1:** List of accessions for simple sequence repeat (SSR) analysis, listed by their variety name or tree number (TN). Variety names in double quotes are internal working names for unidentified varieties in the germplasm collection. Code: Identifies the individual sample in the study; TN in Collection: Identifies the specific tree in the respective collection; Sample Origin: Country-Code where the plant material was collected; Variety Origin: Country-Code of the Country where this variety originated; Sample Set: Accessions belong to one of three sample sets: STB: Stoob, BOK: BOKU, Vienna germplasm collection, IS: International Standards; Interesting traits of Austrian varieties: Some traits of Austrian landraces are listed, that could be interesting for breeders are listed.

Code	Variety Name or TN	TN in Collection	Sample Origin	Variety Origin	No of Trees of this Variety Name	Sample Set	Interesting Traits of Austrian Varieties	Reference
A60	“Bigarreau Burlat”	26113BSch	AT	FR	2	BOK		
A59	“Burlat VG“	26112BVG	AT	FR	1	BOK		
A05	“Butterkirsche”	TN34	AT	AT	5	STB	taste, blushed type, early ripening	[12]
A06	“Butterkirsche”	TN35	AT	AT	5	STB	taste, blushed type, early ripening	[12]
A10	“Butterkirsche”	TN44	AT	AT	5	STB	taste, blushed type, early ripening	[12]
A13	“Butterkirsche”	TN58	AT	AT	5	STB	taste, blushed type, early ripening	[12]
A31	“Butterkirsche”	TN165	AT	AT	5	STB	taste, blushed type, early ripening	[12]
A63	“Chelan”		US	CA	1	IS		
A47	“Donnerskircher Blaukirsche”	2472Do	AT	AT	1	BOK	rich juice color, processed products	
A25	“Early Burlat”		US	FR	1	IS		
A01	“F12/1”		GB	GB	1	IS		
A62	“Fruehe Kirsche Ubl”	26325FKU	AT	AT	1	BOK		
A52	“Frueheste der Mark”	10816FdM	AT	FR	1	BOK	early ripening	[8]
A39	“Germersdorfer”	2428Ge	AT	DE	2	BOK		
A54	“Germersdorfer”	2426T1	AT	DE	2	BOK	fruit size, fruit firmness	
A17	“Goodnestone Black”		GB	GB	1	IS		
A02	“Grosse Schwarze Knorpelkirsche”	TN09	AT	DE	5	STB		
A03	“Grosse Schwarze Knorpelkirsche”	TN29	AT	DE	5	STB		
A04	“Grosse Schwarze Knorpelkirsche”	TN30	AT	DE	5	STB		
A15	“Grosse Schwarze Knorpelkirsche”	TN87	AT	DE	5	STB		
A40	“Grosse Schwarze Knorpelkirsche”	2424GSK	AT	DE	5	BOK		
A14	“Hedelfinger Riesenkirsche”	TN60	AT	DE	5	STB		
A22	“Hedelfinger Riesenkirsche”	TN128	AT	DE	5	STB		
A27	“Hedelfinger Riesenkirsche”	TN146	AT	DE	5	STB		
A38	“Hedelfinger Riesenkirsche”	2444HR	AT	DE	5	BOK		
A55	“Hedelfinger Riesenkirsche”	2437T2	AT	DE	5	BOK		
A23	“Horitschoner Herzkirsche”	TN133	AT	AT	1	STB	fruit size, fruit firmness	
A37	“Horitschoner Herzkirsche”	2452HH	AT	AT	1	BOK	fruit size, fruit firmness	
A51	“Hybrid 222”	10812Hy	AT	FR	1	BOK		
A19	“Jaboulay”	TN109	AT	FR	2	STB	early ripening	
A43	“Jaboulay”	26120Sch	AT	FR	2	BOK	early ripening	
A44	“Kritzendorfer Einsiedekirsche” Type I	26225KrD1	AT	AT	1	BOK	rich juice color, processed products	
A45	“Kritzendorfer Einsiedekirsche” Type II	2641KrD2	AT	AT	1	BOK	rich juice color, processed products	
A46	“Lambert”	2456La	AT	US	1	BOK		
A09	“Lapins”		US	CA	1	IS		
A16	“Marzer Kirsche”	TN95	AT	AT	2	STB	high fruit acidity, high ratio fruit weight/stone weight, early ripening	[8]
A36	“Marzer Kirsche”	2468Ma	AT	AT	2	BOK	high fruit acidity, high ratio fruit weight/stone weight, early ripening	[8]
A42	“Melker Riesenkirsche”	2431MRK	AT	AT	1	BOK	fruit size, fruit firmness	[8]
A33	“Napoleon”		GB	DE	1	IS		
A58	“Noble”		GB	GB	1	IS		
A49	“Noire de Meched”		GB	IR	1	IS		
A41	“NY54”		US	US	1	IS		
A26	“Saemling von Sauerbrunn”	TN143	AT	AT	1	STB	tolerance to fruit cracking	[8]
A61	“Saemling von Sauerbrunn”	26122LaSt	AT	AT	2	BOK	tolerance to fruit cracking	[8]
A48	“Sarga Dragan”	1071SD	AT	HU	1	BOK		
A56	“Schneiders Spaete Knorpelkirsche”		US	DE	2	IS		
A53	“Schneiders Spaete Knorpelkirsche”	2422SSK	AT	DE	2	BOK		
A57	“Stella Spur”	2453StP	AT	CA	1	BOK		
A50	“Tavriczskai”	1082Ta	AT	HU	1	BOK		
A20	TN110	TN110	AT	AT	1	STB	early ripening, mechanical harvest	[12]
A21	TN120	TN120	AT	AT	1	STB	early ripening	[12]
A24	TN142	TN142	AT	AT	1	STB	early ripening, mechanical harvest	[12]
A28	TN155	TN155	AT	AT	1	STB		
A29	TN157	TN157	AT	AT	1	STB		
A30	TN163	TN163	AT	AT	1	STB	mechanical harvest	[12]
A32	TN175	TN175	AT	AT	1	STB	fruit size, fruit firmness	
A34	TN177	TN177	AT	AT	1	STB		
A07	TN36	TN36	AT	AT	1	STB		
A08	TN39	TN39	AT	AT	1	STB		
A11	TN46	TN46	AT	AT	1	STB	mechanical harvest	[12]
A12	TN52	TN52	AT	AT	1	STB	early ripening	[12]
A18	TN96	TN96	AT	AT	1	STB	taste, early ripening	[12]
A35	“Ulster”		US	US	1	IS		

**Table 2:** List of accessions for AFLP-analysis listed by their variety name. Code, TN: identifies the individual tree in the respective sample set for AFLP-analysis. Sample Origin: Country-Code where the plant material was collected, Variety Origin: Country-Code where this variety originated; Sample Set: Accessions for AFLP-analysis belong to one of four sample sets: Leitha: Leithaberg, Scharten, BOK: BOKU, Vienna germplasm collection, IS: International Standards; The column "SSR-analysis (Table 1)” indicates if this tree has also been included in the SSR-analysis presented in this study.

Code, TN	Variety Name or TN	Sample Origin	Variety Origin	Sample Set	SSR-Analysis (Table 1)
2451F12_1	“F12/1”	GB	GB	IS	Yes
2425T1	“Germersdorfer”	AT	DE	BOK	No
2428Ge	“Germersdorfer”	AT	DE	BOK	Yes
Ge_alt_VG	“Germersdorfer”	AT	DE	BOK	No
B04	“Germersdorfer”	AT	DE	Leitha	No
P10	“Germersdorfer”	AT	DE	Leitha	No
S20	“Germersdorfer”	AT	DE	Scharten	No
S26	“Germersdorfer”	AT	DE	Scharten	No
2452HH	“Horitschoner Herzkirsche”	AT	AT	BOK	Yes
D10	“Horitschoner Herzkirsche”	AT	AT	Leitha	No
P12	“Horitschoner Herzkirsche”	AT	AT	Leitha	No
2431MRK	“Melker Riesenkirsche”	AT	AT	BOK	Yes
P09	“Melker Riesenkirsche”	AT	AT	Leitha	No
Noble 2013, Noble 2015	“Noble”	GB	GB	IS	Yes
N.d.M. 2013, N.d.M.2015	“Noire de Meched”	GB	IR	IS	Yes
K02	“Rainkirsche”	AT	AT	Scharten	No
2422SSK	“Schneiders Spaete Knorpelkirsche”	AT	DE	BOK	Yes
SSK_alt_VG	“Schneiders Spaete Knorpelkirsche”	AT	DE	BOK	No
SSKB	“Schneiders Spaete Knorpelkirsche”	AT	DE	Scharten	No

**Table 3:** Primers used for SSR-analysis in multiplex PCR reactions.

Multiplex	Primer	nmol in 13 µL PCR Reaction	Dye	Linkage Group	Reference
MP1	EMPa002	2.50	PET	LG1	[34]
	EMPaS12	1.75	FAM	LG6	[35]
	EMPaS02	3.75	NED	LG3	[35]
	UDP98-412	1.25	VIC	LG6	[36]
	CPPCT006	2.50	PET	LG8	[37]
	EMPaS01	5.00	FAM	LG6	[35]
	EMPa017	5.00	NED	LG2	[34]
	CPPCT022	2.50	VIC	LG7	[37]
MP2	CPSCT038	2.50	NED	LG2	[38]
	BPPCT034	3.00	PET	LG2	[39]
	BPPCT037	1.75	FAM	LG5	[39]
	EMPaS10	1.75	VIC	LG4	[35]

**Table 4:** Genetic parameters detected for SSR markers used to study autochthonous Austrian sweet cherry accessions and international varieties.

Locus	N	Ho	He	PIC	PA	PA-MLGs
BPPCT037	8	0.89	0.81	0.78	2	2
CPPCT006	8	0.89	0.77	0.73	2	2
CPPCT022	8	0.51	0.59	0.56	4	6
CPSCT038	3	0.58	0.61	0.54		
EMPa002	3	0.75	0.54	0.45		
EMPa017	5	0.06	0.23	0.22	1	1
EMPaS01	5	0.73	0.70	0.66		
EMPaS02	7	0.84	0.72	0.68	1	1
EMPaS10	8	0.49	0.60	0.53	4	5
EMPaS12	5	0.83	0.75	0.71		
UDP98-412	9	0.71	0.70	0.67	1	1
Total	69					
Average	6.3	0.66	0.64	0.59		

N, number of alleles per locus; Ho, observed heterozygosity; He, expected heterozygosity; PIC, polymorphic information content; PA, no. of private alleles per locus; PA-MLGs, Number of Multi locus genotypes with private alleles per locus.

**Table 5:** Composition of clusters, membership of Multilocus Genotypes according to DAPC based on SSR data. Column 4 shows the code of the accessions with the same genotype where applicable. STB = Stoob, IS = International Standard, BOK = BOKU, Vienna, TN = Tree number. Variety names in double quotes are internal working names for unidentified varieties in the germplasm collection.

Cluster	Variety	Sample Set	Accessions with Same Genotype	Code
1	“F12/1”	IS		A01
1	“Butterkirsche”	STB	A06, A07, A10, A13, A31	A05
1	TN110	STB		A20
1	TN163	STB		A30
1	“NY54”	IS		A41
1	“Kritzendorfer Einsiedekirsche” “Type I”	BOK	A45	A44
1	“Donnerskircher Blaukirsche”	BOK		A47
1	“Sämling von Sauerbrunn”	BOK		A61
1	“Frühe Kirsche Ubl”	BOK		A62
2	“Große Schwarze Knorpelkirsche”	STB		A02
2	“Große Schwarze Knorpelkirsche”	STB	A04	A03
2	“Lapins”	IS		A09
2	TN46	STB		A11
2	TN52	STB		A12
2	“Hedelfinger Riesenkirsche”	STB	A22	A14
2	“Große Schwarze Knorpelkirsche”	STB		A15
2	“Goodnestone Black”	IS		A17
2	“Sämling von Sauerbrunn”	STB		A26
2	“Hedelfinger Riesenkirsche”	STB		A27
2	“Napoleon”	IS		A33
2	“Ulster”	IS		A35
2	“Hedelfinger Riesenkirsche”	BOK		A38
2	“Große Schwarze Knorpelkirsche”	BOK		A40
2	“Lambert”	BOK	A57	A46
2	“Sarga Dragan”	BOK		A48
2	“Tavriczskai”	BOK		A50
2	“Tscholl 2”	BOK		A55
3	TN39	STB	A23, A28, A29, A32, A34, A37, A39, A42, A49, A53, A54, A56	A08
3	“Marzer Kirsche”	STB		A16
3	TN96	STB	A21, A24	A18
3	“Jaboulay”	STB	A43	A19
3	“Early Burlat”	IS		A25
3	“Marzer Kirsche”	BOK		A36
3	“Hybrid 222”	BOK	A59	A51
3	“Früheste der Mark”	BOK		A52
3	“Noble”	IS		A58
3	“Bigarreau Burlat”	BOK		A60
3	“Chelan”	IS		A63

**Table 6:** Diversity indices for SSR data grouped according to DAPC clusters.

Pop	N	MLG	eMLG	SE	lambda	Hexp	Ia	rbarD
1	9	9	9	0	0.889	0.630	1.035	0.1075
2	18	18	10	5.43 × 10^−7^	0.944	0.595	0.316	0.0331
3	11	11	10	0	0.909	0.614	0.634	0.0654
Total	38	38	10	1.72 × 10^−6^	0.974	0.665	0.653	0.0662

Pop, population from DAPC analysis; N: no. of individuals; MLG: multi-locus-genotypes; eMLG, expected MLGs based on rarefaction; SE, standard error from rarefaction; lambda, Simpson’s index; Hexp, Neis expected heterozygosity; Ia, Index of association; rbarD, standardized index of association.

## Data Availability

The data from SSR- and AFLP-analysis is provided as supplemental files (Supplement S1, Supplement S2) to this manuscript.

## Supplementary Materials

The following are available online at https://www.mdpi.com/2073-4425/12/3/322/s1, Table S1: SSR-data, Table S2: AFLP-data.
