# Supplementary material for: Autochthonous Austrian Varieties of Prunus avium L. Represent a Regional Gene Pool, Assessed Using SSR and AFLP Markers
Source: Genes (Basel). 2021 Feb 24;12(3):322. doi: 10.3390/genes12030322 (PMC7995972; doi:10.3390/genes12030322)
Supplement: Supplementary file 1 [file genes-12-00322-s001.zip › Table S1_SSR-data.pdf]

Supplement 1: for manuscript: Autochthonous Austrian varieties of Prunus avium L. represent a regional gene pool, assessed using SSR and AFLP markers. Schüller et al.: SSR-data

Code, Variety or Tree Number	Sample Set	EMPaS12		EMPaS02		CPPCT006		CPPCT022		UDP98-412		EMPaS01		EMPa017		EMPa002		BPPCT037		CPSCT038		EMPaS10	
A01,'F12/1'	IS	122	144	143	143	182	184	253	253	116	123	220	226	238	242	105	105	148	150	192	192	166	184
A02,'Grosse Schwarze Knorpelkirsche'	STB	138	144	137	146	182	184	245	254	98	118	226	230	237	237	101	103	144	150	190	204	151	151
A03,'Grosse Schwarze Knorpelkirsche'	STB	144	146	134	137	182	200	253	254	98	118	226	228	237	237	101	103	144	144	190	204	151	151
A04,'Grosse Schwarze Knorpelkirsche'	STB	144	146	134	137	182	200	253	254	98	118	226	228	237	237	101	103	144	144	190	204	151	151
A05,'Butterkirsche'	STB	122	138	137	146	182	196	245	245	119	119	220	220	238	238	103	105	137	152	192	204	166	166
A06,'Butterkirsche'	STB	122	138	137	146	182	196	245	245	119	119	220	220	238	238	103	105	137	152	192	204	166	166
A07,TN36	STB	122	138	137	146	182	196	245	245	119	119	220	220	238	238	103	105	137	152	192	204	166	166
A08,TN39	STB	138	144	137	146	184	200	245	245	119	125	226	230	238	238	103	105	139	144	190	190	151	166
A09,'Lapin'	IS	144	144	137	137	184	184	245	253	119	119	226	226	238	238	103	105	139	144	204	204	151	151
A10,'Butterkirsche'	STB	122	138	137	146	182	196	245	245	119	119	220	220	238	238	103	105	137	152	192	204	166	166
A11,TN46	STB	138	138	134	137	182	184	245	255	100	125	226	228	238	238	103	105	133	144	0	0	162	188
A12,TN52	STB	136	144	134	134	196	200	245	253	100	119	226	226	239	239	105	105	144	150	190	204	151	151
A13,'Butterkirsche'	STB	122	138	137	146	182	196	245	245	119	119	220	220	238	238	103	105	137	152	192	204	166	166
A14,'Hedelfinger Riesenkirsche'	STB	138	144	137	146	182	184	245	255	100	119	226	230	238	238	103	105	144	150	190	204	151	151
A15,'Grosse Schwarze Knorpelkirsche'	STB	144	146	134	137	182	200	253	255	100	119	226	228	238	238	103	105	144	144	190	204	151	151
A16,'Marzer Kirsche'	STB	138	144	137	139	184	196	245	245	119	119	230	236	238	238	103	105	137	139	190	204	166	184
A17,'Goodnestone Black'	IS	136	138	129	137	182	184	255	259	100	123	226	228	238	238	103	105	131	144	190	204	164	166
A18,TN96	STB	136	138	137	141	180	184	247	251	116	125	230	236	238	238	105	105	137	150	192	204	151	153
A19,'Jaboulay'	STB	146	146	137	141	184	186	245	253	116	119	226	236	238	238	105	105	137	152	190	192	188	188
A20,TN110	STB	138	144	141	146	182	200	245	245	98	115	226	230	238	238	101	103	150	152	192	192	151	153
A21,TN120	STB	136	138	137	141	180	184	247	251	116	125	230	236	238	238	105	105	137	150	192	204	151	153
A22,'Hedelfinger Riesenkirsche'	STB	138	144	137	146	182	184	245	255	100	119	226	230	238	238	103	105	144	150	190	204	151	151
A23,'Horitschoner Herzkirsche'	STB	138	144	137	146	184	200	245	245	119	125	226	230	238	238	103	105	139	144	190	190	151	166
A24,TN142	STB	136	138	137	141	180	184	247	251	116	125	230	236	238	238	105	105	137	150	192	204	151	153
A25,'Early Burlat'	IS	136	146	134	141	184	184	245	253	116	119	230	236	238	238	105	105	137	139	190	190	151	151
A26,'Saemling von Sauerbrunn'	STB	138	138	137	141	182	182	245	245	119	119	226	226	238	238	103	105	133	150	204	204	151	151
A27,'Hedelfinger Riesenkirsche'	STB	138	138	137	139	182	200	245	255	100	119	228	230	238	238	103	105	144	150	190	204	151	151
A28,TN155	STB	138	144	137	146	184	200	245	245	119	125	226	230	238	238	103	105	139	144	190	190	151	166
A29,TN157	STB	138	144	137	146	184	200	245	245	119	125	226	230	238	238	103	105	139	144	190	190	151	166
A30,TN163	STB	136	136	137	146	182	200	245	245	116	125	220	226	238	238	103	105	133	152	192	192	151	166
A31,'Butterkirsche'	STB	122	138	137	146	182	196	245	245	119	119	220	220	238	238	103	105	137	152	192	204	166	166
A32,TN175	STB	138	144	137	146	184	200	245	245	119	125	226	230	238	238	103	105	139	144	190	190	151	166
A33,'Napoleon'	IS	138	138	137	139	184	200	253	255	119	119	228	228	238	238	103	105	137	144	190	204	151	151
A34,TN177	STB	138	144	137	146	184	200	245	245	119	125	226	230	238	238	103	105	139	144	190	190	151	166
A35,'Ulster'	IS	138	144	137	146	182	184	245	253	119	119	226	226	238	238	103	105	133	144	190	192	151	151
A36,'Marzer Kirsche'	BOK	122	138	137	146	184	200	245	257	119	123	220	236	237	237	105	105	133	139	190	204	151	185
A37,'Horitschoner Herzkirsche'	BOK	138	144	137	146	184	200	245	245	119	125	226	230	238	238	103	105	139	144	190	190	151	166
A38,'Hedelfinger Riesenkirsche'	BOK	144	146	137	141	184	196	245	257	119	119	226	226	238	238	103	105	137	144	190	204	151	151
A39,'Germersdorfer'	BOK	138	144	137	146	184	200	245	245	119	125	226	230	238	238	103	105	139	144	190	190	151	166
A40,'Grosse Schwarze Knorpelkirsche'	BOK	144	146	134	137	182	200	253	255	98	118	226	226	238	238	101	103	144	144	190	204	151	151
A41,'NY54'	IS	146	146	134	134	182	196	255	255	119	119	220	226	238	238	105	105	144	144	190	192	166	166
A42,'Melker Riesenkirsche'	BOK	138	144	137	146	184	200	245	245	119	125	226	230	238	238	103	105	139	144	190	190	151	166
A43,'Jaboulay'	BOK	146	146	137	141	184	186	245	253	116	119	226	236	238	238	105	105	137	152	190	192	188	188

Code, Variety or Tree Number	Sample Set	EMPaS12		EMPaS02		CPPCT006		CPPCT022		UDP98-412		EMPaS01		EMPa017		EMPa002		BPPCT037		CPSCT038		EMPaS10	
A44,'Kritzendorfer Einsiedekirsche' Type I	BOK	136	144	143	146	184	202	253	257	119	125	226	230	238	238	103	105	133	150	190	192	151	166
A45,'Kritzendorfer Einsiedekirsche' Type II	BOK	136	144	143	146	184	202	253	257	119	125	226	230	238	238	103	105	133	150	190	192	151	166
A46,'Lambert'	BOK	144	146	137	146	184	198	245	253	119	119	226	230	238	238	103	105	137	144	190	204	151	151
A47,'Donnerskircher Blaukirsche'	BOK	122	136	137	137	182	196	245	245	116	119	226	230	238	238	103	105	133	152	192	204	151	166
A48,'Sarga Dragan'	BOK	136	144	134	137	196	200	253	253	98	118	226	230	238	238	101	101	144	150	190	204	151	151
A49,'Noire de Meched'	IS	138	144	137	146	184	200	245	245	119	125	226	230	238	238	103	105	139	144	190	190	151	166
A50,'Tavriczskai'	BOK	136	138	137	137	184	196	253	255	100	119	226	230	238	238	103	103	150	150	190	190	151	151
A51,'Hybrid 222'	BOK	138	146	137	137	196	196	245	253	111	119	226	226	232	239	103	105	137	139	190	190	164	166
A52,'Frueheste der Mark'	BOK	136	144	134	134	184	196	245	253	119	125	226	230	238	238	105	105	139	150	190	190	151	151
A53,'Schneiders Spaete Knorpelkirsche'	BOK	138	144	137	146	184	200	245	245	119	125	226	230	238	238	103	105	139	144	190	190	151	166
A54,'Germersdorfer'	BOK	138	144	137	146	184	200	245	245	119	125	226	230	238	238	103	105	139	144	190	190	151	166
A55,'Hedelfinger Riesenkirsche'	BOK	138	138	137	139	182	200	245	255	98	118	228	228	238	238	101	103	144	150	190	204	151	151
A56,Schneiders Spaete Knorpelkirsche	IS	138	144	137	146	184	200	245	245	119	125	226	230	238	238	103	105	139	144	190	190	151	166
A57,'Stella Spur'	BOK	144	146	137	146	184	198	245	253	119	119	226	230	238	238	103	105	137	144	190	204	151	151
A58,'Noble'	IS	138	144	139	143	184	184	245	245	116	116	226	230	232	238	105	105	137	137	190	190	151	166
A59,Burlat VG	BOK	138	146	137	137	196	196	245	253	111	119	226	226	232	239	103	105	137	139	190	190	164	166
A60,'Biggareau Burlat'	BOK	136	146	134	141	184	184	245	245	116	116	230	236	238	238	105	105	137	139	190	190	151	188
A61,'Saemling von Sauerbrunn'	BOK	122	136	143	146	182	200	245	259	116	119	220	230	238	238	103	105	133	152	192	192	151	166
A62,Fruehe Kirsche Ubl*	BOK	136	136	134	134	182	196	245	245	119	119	226	230	238	238	103	105	133	150	190	192	166	166
A63,'Chelan'	IS	136	146	134	146	184	198	245	245	116	119	226	226	238	238	105	105	137	139	190	192	151	151
