# Supplementary material for: Autochthonous Austrian Varieties of Prunus avium L. Represent a Regional Gene Pool, Assessed Using SSR and AFLP Markers
Source: Genes (Basel). 2021 Feb 24;12(3):322. doi: 10.3390/genes12030322 (PMC7995972; doi:10.3390/genes12030322)
Supplement: Supplementary file 1 [file genes-12-00322-s001.zip › Table S2_AFLP-data.pdf]

Supplement 2: for manuscript: Autochthonous Austrian varieties of Prunus avium L. represent a regional gene pool, assessed using SSR and AFLP markers. Schüller et al.: AFLP-data

Sample ID	Sample Set	E12-M19-0378	E12-M19-0346	E12-M19-0342	E12-M19-0318	E12-M19-0315	E12-M19-0311	E12-M19-0298	E12-M19-0286	E12-M19-0284	E12-M19-0281	E12-M19-0265	E12-M19-0244	E12-M19-0242	E12-M19-0235	E12-M19-231	E12-M19-0226	E12-M19-0221	E12-M19-0216	E12-M19-0211	E12-M19-0209	E12-M19-0206	E12-M19-0193	E12-M19-0180	E12-M19-0175	E12-M19-0173	E12-M19-0169	E12-M19-0156	E12-M19-0142	E12-M19-0130	E12-M19-0125	E12-M19-0122	E12-M19-0117	E12-M19-0109	E12-M19-0101	E-AGG-M17-0374	E-AGG-M17-0357	E-AGG-M17-0353	E-AGG-M17-0347	E-AGG-M17-0345	E-AGG-M17-0327	E-AGG-M17-0325	E-AGG-M17-0319	E-AGG-M17-0318	E-AGG-M17-0314	E-AGG-M17-0313	E-AGG-M17-0312	E-AGG-M17-0310	E-AGG-M17-0307	E-AGG-M17-0305	E-AGG-M17-0303	E-AGG-M17-0299
2422SSK	BOK	1	1	1	1	0	1	1	0	0	0	1	0	0	0	0	0	1	1	1	1	1	1	1	1	1	1	0	1	0	1	1	1	0	0	1	1	0	1	1	1	1	1	1	1	1	1	1	1	1	1	0
2425T1	BOK	1	1	1	1	0	1	1	0	0	0	1	0	0	1	0	0	1	1	1	1	1	1	1	1	1	1	0	1	0	1	1	1	1	0	1	1	0	1	1	1	1	1	1	1	1	1	1	1	1	1	0
2426T1	BOK	1	1	1	1	0	1	1	0	0	0	1	0	0	1	0	1	1	1	1	1	1	1	1	1	1	1	0	1	0	1	1	1	0	1	1	1	0	1	1	1	1	1	1	1	1	1	1	1	1	1	0
2428Ge	BOK	1	1	1	1	0	1	1	0	0	0	1	0	0	1	0	1	1	1	1	1	1	1	1	1	1	1	0	1	0	1	1	1	1	1	1	1	0	1	1	1	1	1	1	1	1	1	1	1	1	1	0
2431MRK	BOK	1	1	1	1	1	1	1	0	0	0	1	1	1	1	0	1	1	1	1	1	1	1	1	1	1	1	0	1	0	1	1	1	1	0	1	1	0	1	1	1	1	1	1	1	1	1	1	1	1	1	0
2431MRK	BOK	1	1	1	1	0	1	1	0	0	0	1	0	0	1	0	1	1	1	1	1	1	1	1	1	1	1	0	1	0	1	1	1	1	1	1	1	0	1	1	1	1	1	1	1	1	1	1	1	1	1	0
2431MRK	BOK	1	1	1	1	0	1	1	0	0	0	1	0	0	1	0	1	1	1	1	1	1	1	1	1	1	1	0	1	0	1	1	1	1	0	1	1	0	1	1	1	1	1	1	1	1	1	1	1	1	1	0
2451F12_1	BOK	1	1	1	1	0	1	1	0	0	0	1	0	0	1	1	1	1	1	1	1	1	1	1	1	1	1	0	1	0	1	1	1	1	1	1	1	0	1	1	1	1	1	1	1	1	1	1	1	1	1	0
2451F12_1	BOK	1	1	1	1	0	1	1	0	0	0	1	1	0	1	1	1	1	1	1	1	1	1	1	1	1	0	0	1	1	1	1	1	1	1	1	1	0	1	0	1	1	1	1	1	1	1	1	1	1	1	1
2452HH	BOK	1	1	1	1	0	1	1	0	0	0	1	0	0	0	0	1	1	1	1	1	1	1	1	1	1	1	0	1	0	1	1	1	1	1	1	1	0	1	1	1	1	1	1	1	1	1	1	1	1	1	0
2452HH	BOK	1	1	1	1	0	1	1	0	0	0	1	0	0	0	0	1	1	1	1	1	1	1	1	1	1	1	0	1	0	1	1	1	1	1	1	1	1	1	1	1	1	1	1	1	1	1	1	1	1	1	0
2452HH	BOK	1	1	1	1	0	1	1	0	0	0	1	0	0	0	0	1	1	1	1	1	1	1	1	1	1	1	0	1	0	1	1	1	1	1	1	1	1	1	1	1	1	1	1	1	1	1	1	1	1	1	0
Ge_alt_VG	BOK	1	1	1	1	0	1	1	0	0	0	1	0	0	0	0	1	1	1	1	1	1	1	1	1	1	1	0	1	0	1	1	1	1	1	1	1	1	1	1	1	1	1	1	1	1	1	1	1	1	1	0
SSK_alt_VG	BOK	1	1	1	1	0	1	1	1	1	1	1	0	0	1	0	1	1	1	1	1	1	1	1	1	1	1	0	1	0	1	1	1	1	1	1	1	1	1	1	1	1	1	1	1	1	1	1	1	1	1	0
SSK_alt_VG	BOK	1	1	1	1	0	1	1	1	1	1	1	0	0	1	0	1	1	1	1	1	1	1	1	1	1	1	0	1	0	1	1	1	1	1	1	1	0	1	1	1	1	1	1	1	1	1	1	1	1	1	0
N.d.M. 2015	INT	1	1	1	1	0	1	1	0	0	0	1	0	0	1	0	1	1	1	1	1	1	1	1	1	1	1	0	1	0	1	1	1	0	1	1	1	0	1	1	1	1	1	1	1	1	1	1	1	1	1	0
Noble 2013	INT	1	1	1	1	0	1	1	0	0	0	1	0	0	0	0	1	1	1	1	1	1	1	1	1	1	1	0	1	0	1	1	1	0	1	1	1	0	1	0	1	1	1	1	1	1	1	1	1	1	1	0
N.d.M.2013	INT	1	1	1	1	0	1	1	0	0	0	1	0	0	1	0	0	1	1	0	1	1	0	1	1	1	1	0	1	0	1	1	1	0	1	1	1	0	1	1	1	1	1	1	1	1	1	0	0	0	1	0
Noble 2015	INT	1	1	0	1	0	1	1	0	0	0	1	0	0	0	0	0	1	1	0	1	1	0	1	1	1	1	0	1	0	1	1	1	0	1	1	1	0	1	-1	1	1	1	1	1	-1	0	0	0	0	1	0
B04	LEIT	1	1	1	1	0	1	1	0	0	0	1	0	0	0	0	1	1	1	1	1	1	1	1	1	1	1	0	1	0	1	1	1	0	1	1	1	0	1	1	1	1	1	1	1	1	1	1	1	1	1	0
B04	LEIT	1	1	1	1	0	1	1	0	0	0	1	0	0	0	0	1	1	1	1	1	1	1	1	1	1	1	0	1	0	1	1	1	0	0	1	1	0	1	1	1	1	1	1	1	1	1	1	1	1	1	0
D10	LEIT	1	1	1	1	0	1	1	0	0	0	1	0	0	1	0	1	1	1	1	1	1	1	1	1	1	1	0	1	0	1	1	1	0	0	1	1	0	1	1	1	1	1	1	1	1	1	1	1	1	1	0
D10	LEIT	1	1	1	1	0	1	1	0	0	0	1	0	0	1	0	1	1	1	1	1	1	1	1	1	1	1	0	1	0	1	1	1	0	1	1	1	1	1	1	1	1	1	1	1	1	1	1	1	1	1	0
P09	LEIT	1	1	1	1	0	1	1	0	0	0	1	0	0	0	0	1	1	1	1	1	1	1	1	1	1	1	0	1	0	1	1	1	0	1	1	1	0	1	1	1	1	1	1	1	1	1	1	1	1	1	0
P09	LEIT	1	1	1	1	0	1	1	1	1	1	1	0	0	1	0	1	1	1	1	1	1	1	1	1	1	1	0	1	0	1	1	1	0	1	1	1	1	1	1	1	1	1	1	1	1	1	1	1	1	1	0
P10	LEIT	1	1	1	1	0	1	1	1	1	1	1	0	0	1	0	1	1	1	1	1	1	1	1	1	1	1	0	1	0	1	1	1	0	1	1	0	1	1	1	1	1	1	1	1	1	1	1	1	1	1	0
P10	LEIT	1	1	1	1	0	1	1	0	0	0	1	0	0	1	0	1	1	1	1	1	1	1	1	1	1	1	0	1	0	1	1	1	0	1	1	0	1	1	1	1	1	1	1	1	1	1	1	1	1	1	0
P12	LEIT	1	1	1	1	0	1	1	0	0	0	1	0	0	1	0	1	1	1	1	1	1	1	1	1	1	1	0	1	0	1	1	1	0	1	1	0	1	1	1	1	1	1	1	1	1	1	1	1	1	1	0
S20	SCH	1	1	1	1	0	1	1	0	0	0	1	0	0	1	0	1	1	1	1	1	1	1	1	1	1	1	0	1	0	1	1	1	0	1	1	1	1	1	1	1	1	1	1	1	1	1	1	1	1	1	0
S26	SCH	1	1	1	1	0	1	1	0	0	0	1	0	0	1	0	1	1	1	1	1	1	1	1	1	1	1	1	1	0	1	1	1	0	1	1	1	0	1	1	1	1	1	1	1	1	1	1	1	1	1	0
S26	SCH	1	1	1	1	0	1	1	1	1	1	1	0	0	1	0	1	1	1	1	1	1	1	1	1	1	1	0	1	0	1	1	1	0	1	1	1	1	1	1	1	1	1	1	1	1	1	1	1	1	1	0
SSKB	SCH	1	1	1	1	0	1	1	0	0	0	1	0	0	1	0	1	1	1	1	1	1	1	1	1	1	1	0	1	0	1	1	1	0	1	1	1	0	1	1	1	1	1	1	1	1	1	1	1	1	1	0
SSKB	SCH	1	1	1	1	0	1	1	0	0	0	1	0	0	1	0	1	1	1	1	1	1	1	1	1	1	1	0	1	0	1	1	1	0	0	1	1	0	1	1	1	1	1	1	1	1	1	1	1	1	1	0
K02	SCH	1	1	1	1	0	1	1	0	0	0	1	0	0	1	0	1	1	1	1	1	1	1	1	1	1	1	0	1	0	1	1	1	0	1	1	1	0	1	1	1	1	1	1	1	1	1	1	1	1	1	0
K02	SCH	1	1	1	1	0	1	1	0	0	0	1	0	0	1	0	1	1	1	1	1	1	1	1	1	1	1	0	1	0	1	1	1	0	1	1	1	0	1	1	1	1	1	1	1	1	1	1	1	1	1	0
K02	SCH	1	1	1	1	0	1	1	0	0	0	1	0	0	1	0	1	1	1	1	1	1	1	1	1	1	1	0	1	0	1	1	1	0	1	1	1	0	1	1	1	1	1	1	1	1	1	1	1	1	1	0

Supplement 2: for manuscrip

Sample ID	Sample Set	E-AGG-M17-0285	E-AGG-M17-0282	E-AGG-M17-0279	E-AGG-M17-0275	E-AGG-M17-0252	E-AGG-M17-0243	E-AGG-M17-0240	E-AGG-M17-0231	E-AGG-M17-0223	E-AGG-M17-0218	E-AGG-M17-0201	E-AGG-M17-0196	E-AGG-M17-0186	E-AGG-M17-0184	E-AGG-M17-0180	E-AGG-M17-0175	E-AGG-M17-0165	E-AGG-M17-0121	E12-M16-0551	E12-M16-0409	E12-M16-0397	E12-M16-0391	E12-M16-0378	E12-M16-0336	E12-M16-0333	E12-M16-0319	E12-M16-0317	E12-M16-0312	E12-M16-0303	E12-M16-0300	E12-M16-0295	E12-M16-0294	E12-M16-0282	E12-M16-0277	E12-M16-0267	E12-M16-0265	E12-M16-0264	E12-M16-0260	E12-M16-0257	E12-M16-0255	E12-M16-0243	E12-M16-0237	E12-M16-0235	E12-M16-0234	E12-M16-0232	E12-M16-0231	E12-M16-0230	E12-M16-0229	E12-M16-0228	E12-M16-0227	E12-M16-0226
2422SSK	BOK	1	1	0	1	1	1	1	1	1	1	0	1	1	1	1	1	1	1	1	1	1	1	1	1	1	1	1	1	1	1	1	1	1	1	1	1	1	1	1	1	0	0	0	0	1	1	1	1	1	0	0
2425T1	BOK	1	1	0	1	1	1	1	1	1	1	0	1	1	1	1	1	1	1	1	1	1	1	1	1	1	1	1	1	1	1	1	1	1	1	1	1	1	1	1	1	0	0	0	0	1	1	1	1	1	0	0
2426T1	BOK	1	1	0	1	1	1	1	1	1	1	0	1	1	1	1	1	1	1	1	1	1	1	1	1	1	1	1	1	1	1	1	1	1	1	1	1	1	1	1	1	1	0	0	0	1	1	1	1	1	0	0
2428Ge	BOK	1	1	0	1	1	1	1	1	1	1	0	1	1	1	1	1	1	1	1	1	1	1	1	1	1	1	1	1	1	1	1	1	1	1	1	1	1	1	1	1	1	0	0	0	1	1	1	1	1	0	0
2431MRK	BOK	1	1	0	1	1	1	1	1	1	1	0	1	1	1	1	1	1	1	1	1	1	1	1	1	1	1	1	1	1	1	1	1	1	1	1	1	1	1	1	1	1	0	0	0	1	1	1	1	1	0	0
2431MRK	BOK	1	1	0	1	1	1	1	1	1	1	0	1	1	1	1	1	1	1	1	1	1	1	1	1	1	1	1	1	1	1	1	1	1	1	1	1	1	1	1	1	1	0	0	0	1	1	1	1	1	0	0
2431MRK	BOK	1	1	0	1	1	1	1	1	1	1	0	1	1	1	1	1	1	1	1	1	1	1	1	1	1	1	1	1	1	1	1	1	1	1	1	1	1	1	1	1	1	0	0	0	1	1	1	1	1	0	0
2451F12_1	BOK	1	1	0	1	1	1	1	1	1	1	0	1	1	1	1	1	1	1	1	1	1	1	1	1	1	1	1	1	1	1	1	1	1	1	1	1	1	1	1	1	1	0	0	0	1	1	1	1	1	0	0
2451F12_1	BOK	1	1	0	1	1	1	1	1	1	1	0	1	1	0	1	1	1	1	1	1	1	0	1	1	1	1	1	1	1	1	1	1	1	1	1	1	1	1	0	1	0	0	0	0	1	1	1	1	1	0	0
2452HH	BOK	1	1	0	1	1	1	1	1	1	1	0	1	1	1	1	1	1	1	1	1	1	1	1	-1	1	1	1	1	1	1	1	1	1	1	1	1	1	1	1	1	1	0	0	0	1	1	1	1	1	0	0
2452HH	BOK	1	1	0	1	1	1	1	1	1	1	0	1	1	1	1	1	1	1	1	1	1	1	1	1	1	1	1	1	1	1	1	1	1	1	1	1	1	1	1	1	1	0	0	0	1	1	1	1	1	0	0
2452HH	BOK	1	1	0	1	1	1	1	1	1	1	0	1	1	1	1	1	1	1	1	1	1	1	1	1	1	1	1	1	1	1	1	1	1	1	1	1	1	1	1	1	1	0	0	0	1	1	1	1	1	0	0
Ge_alt_VG	BOK	1	1	0	1	1	1	1	1	1	1	0	1	1	1	1	1	1	1	1	1	1	1	1	1	1	1	1	1	1	1	1	1	1	1	1	1	1	1	1	1	1	0	0	0	1	1	1	1	1	0	0
SSK_alt_VG	BOK	1	1	0	1	1	1	1	1	1	1	1	1	1	1	1	1	1	1	1	1	1	1	1	1	1	1	1	1	1	1	1	1	1	1	1	1	1	1	1	1	1	0	0	0	1	1	1	1	1	0	0
SSK_alt_VG	BOK	1	1	0	1	1	1	1	1	1	1	1	1	1	1	1	1	1	1	1	1	1	1	1	1	1	1	1	1	1	1	1	1	1	1	1	1	1	1	1	1	1	0	0	0	1	1	1	1	1	0	0
N.d.M. 2015	INT	1	1	0	1	1	1	1	1	1	1	0	1	1	1	1	1	1	1	1	1	1	1	1	1	1	1	1	1	1	1	1	1	1	1	1	1	1	1	1	1	1	0	0	0	1	1	1	1	1	0	0
Noble 2013	INT	1	1	0	1	1	1	1	1	1	1	1	1	1	1	1	1	1	1	1	1	1	1	1	1	1	1	1	1	1	1	1	1	1	1	1	1	1	1	1	1	1	0	0	0	1	1	1	1	1	0	0
N.d.M.2013	INT	1	1	1	1	1	1	1	1	1	1	0	1	1	1	1	1	1	1	1	1	1	1	1	1	1	1	1	1	1	1	1	1	1	1	1	1	1	1	1	1	1	0	0	0	1	1	1	1	1	0	0
Noble 2015	INT	1	1	1	1	1	1	1	1	1	1	0	1	1	1	1	1	1	1	1	1	1	1	0	1	1	0	0	1	1	1	1	1	1	1	1	1	1	1	1	1	1	0	0	0	1	1	1	1	1	0	0
B04	LEIT	1	1	0	1	1	1	1	1	1	1	0	1	1	1	1	1	1	1	1	1	1	1	1	1	1	1	1	1	1	1	1	1	1	1	1	1	1	1	1	1	1	0	0	0	1	1	1	1	1	0	0
B04	LEIT	1	1	0	1	1	1	1	1	1	1	0	1	1	1	1	1	1	1	1	1	1	1	1	1	1	1	1	1	1	1	1	1	1	1	1	1	1	1	1	1	1	0	0	0	1	1	1	1	1	0	0
D10	LEIT	1	1	0	1	1	1	1	1	1	1	0	1	1	1	1	1	1	1	1	1	1	1	1	1	1	1	1	1	1	1	1	1	1	1	1	1	1	1	1	1	1	0	0	0	1	1	1	1	1	0	0
D10	LEIT	1	1	0	1	1	1	1	1	1	1	0	1	1	1	1	1	1	1	1	1	1	1	1	1	1	1	1	1	1	1	1	1	1	1	1	1	1	1	1	1	1	0	0	0	1	1	1	1	1	0	0
P09	LEIT	1	1	0	1	1	1	1	1	1	1	0	1	1	1	1	1	1	1	1	1	1	1	1	1	1	1	1	1	1	1	1	1	1	1	1	1	1	1	1	1	1	0	0	0	1	1	1	1	1	0	0
P09	LEIT	1	1	0	1	1	1	1	1	1	1	0	1	1	1	1	1	1	1	1	1	1	1	1	1	1	1	1	1	1	1	1	1	1	1	1	1	1	1	1	1	1	0	0	0	1	1	1	1	1	0	0
P10	LEIT	1	1	0	1	1	1	1	1	1	1	0	1	1	1	1	1	1	1	1	1	1	1	1	1	1	1	1	1	1	1	1	1	1	1	1	1	1	1	1	1	1	0	0	0	1	1	1	1	1	0	0
P10	LEIT	1	1	0	1	1	1	1	1	1	1	0	1	1	1	1	1	1	1	1	1	1	1	1	1	1	1	1	1	1	1	1	1	1	1	1	1	1	1	1	1	1	0	0	0	1	1	1	1	1	0	0
P12	LEIT	1	1	0	1	1	1	1	1	1	1	0	1	1	1	1	1	1	1	1	1	1	1	1	1	1	1	1	1	1	1	1	1	1	1	1	1	1	1	1	1	1	0	0	0	1	1	1	1	1	0	0
S20	SCH	1	1	0	1	1	1	1	1	1	1	0	1	1	1	1	1	1	1	1	1	1	1	1	1	1	1	1	1	1	1	1	1	1	1	1	1	1	1	1	1	1	0	0	0	1	1	1	1	1	0	0
S26	SCH	1	1	1	1	1	1	1	1	1	1	1	1	1	1	1	1	1	1	1	1	1	1	1	1	1	1	1	1	1	1	1	1	1	1	1	1	1	1	1	1	1	0	0	0	1	1	1	1	1	0	0
S26	SCH	1	1	0	1	1	1	1	1	1	1	0	1	1	1	1	1	1	1	1	1	1	1	1	1	1	1	1	1	1	1	1	1	1	1	1	1	1	1	1	1	1	0	0	0	1	1	1	1	1	0	0
SSKB	SCH	1	1	0	1	1	1	1	1	1	1	0	1	1	1	1	1	1	1	1	1	1	1	1	1	1	1	1	1	1	1	1	1	1	1	1	1	1	1	1	1	1	0	0	0	1	1	1	1	1	0	0
SSKB	SCH	1	1	0	1	1	1	1	1	1	1	0	1	1	1	1	1	1	1	1	1	1	1	1	1	1	1	1	1	1	1	1	1	1	1	1	1	1	1	1	1	1	0	0	0	1	1	1	1	1	0	0
K02	SCH	1	1	0	1	1	1	1	1	1	1	0	1	1	1	1	1	1	1	1	1	1	1	1	1	1	1	1	1	1	1	1	1	1	1	1	1	1	1	1	1	1	0	0	1	1	1	1	1	1	1	1
K02	SCH	1	1	0	1	1	1	1	1	1	1	0	1	1	1	1	1	1	1	1	1	1	1	1	1	1	1	1	1	1	1	1	1	1	1	1	1	1	1	1	1	0	0	0	1	1	1	1	1	1	1	1
K02	SCH	1	1	0	1	1	1	1	1	1	1	0	1	1	1	1	1	1	1	1	1	1	1	1	1	1	1	1	1	1	1	1	1	1	1	1	1	1	1	1	1	1	0	0	0	1	1	1	1	1	0	0

Supplement 2: for manuscrip

Sample ID	Sample Set	E12-M16-0219	E12-M16-0212	E12-M16-0207	E12-M16-0196	E12-M16-0194	E12-M16-0186	E12-M16-0172	E12-M16-0164	E12-M16-0160	E12-M16-0156	E12-M16-0152	E12-M16-0150	E12-M16-0146	E12-M16-0134	E12-M16-0131	E12-M16-0123	E12-M16-0105	E-AGG-CAG-0365	E-AGG-CAG-0361	E-AGG-CAG-0354	E-AGG-CAG-0346	E-AGG-CAG-0340	E-AGG-CAG-0317	E-AGG-CAG-0313	E-AGG-CAG-0306	E-AGG-CAG-0303	E-AGG-CAG-0301	E-AGG-CAG-0284	E-AGG-CAG-0281	E-AGG-CAG-0273	E-AGG-CAG-0264	E-AGG-CAG-0246	E-AGG-CAG-0235	E-AGG-CAG-0233	E-AGG-CAG-0229	E-AGG-CAG-0218	E-AGG-CAG-0215	E-AGG-CAG-0210	E-AGG-CAG-0201	E-AGG-CAG-0194	E-AGG-CAG-0189	E-AGG-CAG-0177	E-AGG-CAG-0170	E-AGG-CAG-0167	E-AGG-CAG-0161	E-AGG-CAG-0158	E-AGG-CAG-0153	E-AGG-CAG-0149	E-AGG-CAG-0132	E-AGG-CAG-0122	E-AGG-CAG-0112
2422SSK	BOK	1	1	1	1	1	1	1	1	1	1	1	1	0	1	1	1	0	1	1	1	1	1	1	1	1	1	1	1	0	1	1	1	1	0	1	1	1	1	0	1	0	1	0	0	1	1	1	1	1	0	0
2425T1	BOK	1	1	1	1	1	1	1	1	1	1	1	1	0	1	1	1	0	1	1	1	1	1	1	1	1	1	1	1	0	1	1	1	1	0	1	1	1	1	0	1	0	1	1	0	1	1	1	1	1	0	0
2426T1	BOK	1	1	1	1	1	1	1	1	1	1	1	1	0	1	1	1	0	1	1	1	1	1	1	1	1	1	1	1	0	1	1	1	1	1	1	1	1	1	0	1	0	1	1	0	1	1	1	1	1	1	0
2428Ge	BOK	1	1	1	1	1	1	1	1	1	1	1	1	0	1	1	1	0	1	1	1	1	1	1	1	1	1	1	1	0	1	1	1	1	0	1	1	1	1	0	1	0	1	1	0	1	1	1	1	1	1	0
2431MRK	BOK	1	1	1	1	1	1	1	1	1	1	1	1	0	1	1	1	0	1	1	1	1	1	1	1	1	1	1	1	0	1	1	1	1	0	1	1	1	1	0	1	0	1	1	0	1	1	0	1	1	1	0
2431MRK	BOK	1	1	1	1	1	1	1	1	1	1	1	1	0	1	1	1	0	1	1	1	1	1	1	1	1	1	1	1	0	1	1	1	1	0	1	1	1	1	0	1	0	1	1	0	1	1	0	1	1	1	0
2431MRK	BOK	1	1	1	1	1	1	1	1	1	1	1	1	0	1	1	1	0	1	1	1	1	1	1	1	1	1	1	1	0	1	1	1	1	0	1	1	1	1	0	1	0	1	1	0	1	1	0	1	1	1	0
2451F12_1	BOK	1	1	1	1	1	1	1	1	1	1	1	1	0	1	1	1	0	1	1	1	1	1	1	1	1	1	1	1	0	1	1	1	1	0	1	1	1	1	0	1	1	1	0	0	1	1	0	1	1	1	0
2451F12_1	BOK	1	1	1	1	1	1	1	1	1	1	1	1	0	1	1	1	0	1	1	1	1	1	1	1	1	1	1	1	0	1	1	1	1	0	1	1	1	1	0	1	0	1	1	1	1	0	0	1	1	0	0
2452HH	BOK	1	1	1	1	1	1	1	1	1	1	1	1	0	1	1	1	0	1	1	1	1	1	1	1	1	1	1	1	0	1	1	1	1	1	1	1	1	1	1	1	0	1	1	0	1	1	1	1	1	1	0
2452HH	BOK	1	1	1	1	1	1	1	1	1	1	1	1	0	1	1	1	0	1	1	1	1	1	1	1	1	1	1	1	1	1	1	1	1	1	1	1	1	1	0	1	0	1	1	0	1	1	1	1	1	1	0
2452HH	BOK	1	1	1	1	1	1	1	1	1	1	1	1	0	1	1	1	0	1	1	1	1	1	1	1	1	1	1	1	0	1	1	1	1	1	0	1	1	1	0	1	0	1	1	0	1	1	1	1	1	1	0
Ge_alt_VG	BOK	1	1	1	1	1	1	1	1	1	1	1	1	0	1	1	1	0	1	1	1	1	1	1	1	1	1	1	1	1	1	1	1	1	1	1	1	1	1	0	1	0	1	1	0	1	1	1	1	1	1	0
SSK_alt_VG	BOK	1	1	1	1	1	1	1	1	1	1	1	1	0	1	1	1	0	1	1	1	1	1	1	1	1	1	1	1	0	1	1	1	1	1	1	1	1	1	0	1	0	1	1	0	1	1	1	1	1	1	0
SSK_alt_VG	BOK	1	1	1	1	1	1	1	1	1	1	1	1	0	1	1	1	0	1	1	1	1	1	1	1	1	1	1	1	0	1	1	1	1	1	1	1	1	1	0	1	0	1	1	0	1	1	1	1	1	1	0
N.d.M. 2015	INT	1	1	1	1	1	1	1	1	1	1	1	1	0	1	1	1	0	1	1	1	1	1	1	1	1	1	1	1	1	1	1	1	1	1	1	1	1	1	0	1	0	1	0	0	1	1	1	1	1	1	0
Noble 2013	INT	1	1	1	1	1	1	1	1	1	1	1	1	0	1	1	1	0	1	1	1	1	1	1	1	1	1	1	1	0	1	1	1	1	1	1	1	1	1	0	1	0	1	0	0	1	1	1	1	1	1	0
N.d.M.2013	INT	1	1	1	1	1	1	1	1	1	1	1	1	0	1	1	1	1	0	1	1	1	1	1	1	1	1	1	1	0	1	1	1	1	1	1	1	1	1	0	1	0	1	0	1	1	0	0	1	1	0	0
Noble 2015	INT	1	1	1	1	1	1	1	1	1	1	1	1	0	1	1	1	1	0	1	1	1	1	1	1	1	1	1	1	0	1	1	1	1	0	1	1	1	1	0	1	0	1	0	1	1	0	0	1	1	0	0
B04	LEIT	1	1	1	1	1	1	1	1	1	1	1	1	0	1	1	1	0	1	1	1	1	1	1	1	1	1	1	1	0	1	1	1	1	1	1	1	1	1	0	1	1	1	0	0	1	1	1	1	1	1	0
B04	LEIT	1	1	1	1	1	1	1	1	1	1	1	1	0	1	1	1	0	1	1	1	1	1	1	1	1	1	1	1	0	1	1	1	1	1	1	1	1	1	0	1	0	1	0	0	1	1	1	1	1	1	0
D10	LEIT	1	1	1	1	1	1	1	1	1	1	1	1	0	1	1	1	0	1	1	1	1	1	1	1	1	1	1	1	0	1	1	1	1	0	1	1	1	1	0	1	0	1	0	0	1	1	1	1	1	0	0
D10	LEIT	1	1	1	1	1	1	1	1	1	1	1	1	0	1	1	1	0	1	1	1	1	1	1	1	1	1	1	1	0	1	1	1	1	1	1	1	1	1	0	1	0	1	0	0	1	1	1	1	1	1	0
P09	LEIT	1	1	1	1	1	1	1	1	1	1	1	1	0	1	1	1	0	1	1	1	1	1	1	1	1	1	1	1	0	1	1	1	1	1	1	1	1	1	0	1	0	1	0	0	1	1	1	1	1	1	0
P09	LEIT	1	1	1	1	1	1	1	1	1	1	1	1	0	1	1	1	0	1	1	1	1	1	1	1	1	1	1	1	1	1	1	1	1	1	1	1	1	1	0	1	0	1	0	0	1	1	1	1	1	1	0
P10	LEIT	1	1	1	1	1	1	1	1	1	1	1	1	0	1	1	1	0	1	1	1	1	1	1	1	1	1	1	1	0	1	1	1	1	1	1	1	1	1	0	1	0	1	0	0	1	1	1	1	1	1	0
P10	LEIT	1	1	1	1	1	1	1	1	1	1	1	1	0	1	1	1	0	1	1	1	1	1	1	1	1	1	1	1	0	1	1	1	1	1	1	1	1	1	0	1	0	1	0	0	1	1	1	1	1	1	0
P12	LEIT	1	1	1	1	1	1	1	1	1	1	1	1	0	1	1	1	0	1	1	1	1	1	1	1	1	1	1	1	0	1	1	1	1	1	1	1	1	1	0	1	0	1	0	0	1	1	1	1	1	1	0
S20	SCH	1	1	1	1	1	1	1	1	1	1	1	1	0	1	1	1	0	1	1	1	1	1	1	1	1	1	1	1	0	1	1	1	1	0	1	1	1	1	0	1	0	1	0	0	1	1	0	1	1	0	0
S26	SCH	1	1	1	1	1	1	1	1	1	1	1	1	1	1	1	1	0	1	1	1	1	1	1	1	1	1	1	1	0	1	1	1	1	1	1	1	1	1	0	1	1	1	0	0	1	1	1	1	1	1	1
S26	SCH	1	1	1	1	1	1	1	1	1	1	1	1	0	1	1	1	0	1	1	1	1	1	1	1	1	1	1	1	0	1	1	1	1	1	1	1	1	1	0	1	0	1	0	0	1	1	1	1	1	1	0
SSKB	SCH	1	1	1	1	1	1	1	1	1	1	1	1	0	1	1	1	0	1	1	1	1	1	1	1	1	1	1	1	0	1	1	1	1	1	1	1	1	1	0	1	0	1	0	0	1	1	1	1	1	1	0
SSKB	SCH	1	1	1	1	1	1	1	1	1	1	1	1	0	1	1	1	0	1	1	1	1	1	1	1	1	1	1	1	0	1	1	1	1	0	1	1	1	1	0	1	0	0	0	0	1	1	0	1	1	1	0
K02	SCH	1	1	1	1	1	1	1	1	1	1	1	1	0	1	1	1	0	1	1	1	1	1	1	1	1	1	1	1	0	1	1	1	1	1	1	1	1	1	1	1	0	1	0	0	1	1	1	1	1	0	0
K02	SCH	1	1	1	1	1	1	1	1	1	1	1	1	0	1	1	1	0	1	1	1	1	1	1	1	1	1	1	1	0	1	-1	1	1	1	1	1	1	1	1	1	0	1	0	0	1	1	1	1	1	0	0
K02	SCH	1	1	1	1	1	1	1	1	1	1	1	1	0	1	1	1	0	1	1	1	1	1	1	1	1	1	1	1	1	1	1	1	1	1	1	1	1	1	1	1	0	1	0	0	1	1	1	1	1	0	0

Supplement 2: for manuscrip

Sample ID	Sample Set	E16-M17-0430	E16-M17-0422	E16-M17-0404	E16-M17-0399	E16-M17-0392	E16-M17-0383	E16-M17-0374	E16-M17-0366	E16-M17-0331	E16-M17-0314	E16-M17-0307	E16-M17-0300	E16-M17-0261	E16-M17-0234	E16-M17-0219	E16-M17-0200	E16-M17-0195	E16-M17-0189	E16-M17-0129	E-ATA-M17-0465	E-ATA-M17-0447	E-ATA-M17-0373	E-ATA-M17-0363	E-ATA-M17-0351	E-ATA-M17-0338	E-ATA-M17-0320	E-ATA-M17-0310	E-ATA-M17-0294	E-ATA-M17-0288	E-ATA-M17-0281	E-ATA-M17-0273	E-ATA-M17-0262	E-ATA-M17-0246	E-ATA-M17-0239	E-ATA-M17-225	E-ATA-M17-0204	E-ATA-M17-0195	E-ATA-M17-0190	E-ATA-M16-0489	E-ATA-M16-0390	E-ATA-M16-0385	E-ATA-M16-0376	E-ATA-M16-0342	E-ATA-M16-0338	E-ATA-M16-0286	E-ATA-M16-0270	E-ATA-M16-0249	E-ATA-M16-0238	E-ATA-M16-0225	E-ATA-M16-0222	E-ATA-M16-0218
2422SSK	BOK	1	1	1	1	1	1	1	0	1	1	1	1	1	1	1	1	1	1	1	1	1	1	1	1	0	1	1	0	1	1	1	1	0	1	1	1	1	1	1	1	1	1	1	1	1	1	0	1	1	1	1
2425T1	BOK	1	1	1	1	1	1	1	0	1	1	1	1	1	1	1	1	1	1	1	1	1	1	1	1	0	1	1	0	1	1	1	1	0	1	1	1	1	1	1	1	1	1	1	1	1	1	0	1	1	1	1
2426T1	BOK	1	1	1	1	1	1	1	0	1	1	1	1	1	1	1	1	1	1	1	1	1	1	1	1	0	1	1	0	1	1	1	1	0	1	1	1	1	1	1	1	1	1	1	1	1	1	0	1	1	1	1
2428Ge	BOK	1	1	1	1	1	1	1	0	1	1	1	1	1	1	1	1	1	1	1	1	1	1	1	1	0	1	1	0	1	1	1	1	0	1	1	1	1	1	1	1	1	1	1	1	1	1	0	1	1	1	1
2431MRK	BOK	1	1	1	1	1	1	1	0	1	1	1	1	1	1	1	1	1	1	1	1	1	1	1	1	0	1	1	0	1	1	1	1	0	1	1	1	1	1	1	1	1	1	1	1	1	1	0	1	1	1	1
2431MRK	BOK	1	1	1	1	1	1	1	0	1	1	1	1	1	1	1	1	1	1	1	1	1	1	1	1	0	1	1	0	1	1	1	1	0	1	1	1	1	1	1	1	1	1	1	1	1	1	0	1	1	1	1
2431MRK	BOK	1	1	1	1	1	1	1	0	1	1	1	1	1	1	1	1	1	1	1	1	1	1	1	1	0	1	1	0	1	1	1	1	0	1	1	1	1	1	1	1	1	1	1	1	1	1	0	1	1	1	1
2451F12_1	BOK	1	1	1	1	1	1	1	0	1	1	1	1	1	1	1	1	1	1	1	1	1	1	1	1	1	1	1	0	1	1	1	1	1	1	1	1	1	1	1	1	1	1	1	1	1	1	0	1	1	1	1
2451F12_1	BOK	1	1	1	1	1	1	1	0	1	1	1	1	1	1	1	1	1	1	1	1	1	1	1	1	1	1	1	0	1	1	1	1	1	1	1	1	1	1	1	1	1	1	1	1	1	1	0	1	1	1	1
2452HH	BOK	1	1	1	1	1	1	1	0	1	1	1	1	1	1	1	1	1	1	1	1	1	1	1	1	0	1	1	1	1	1	1	1	0	1	1	1	1	1	1	1	1	1	1	1	1	1	0	1	1	1	1
2452HH	BOK	1	1	1	1	1	1	1	0	1	1	1	1	1	1	1	1	1	1	1	1	1	1	1	1	0	1	1	1	1	1	1	1	0	1	1	1	1	1	1	1	1	1	1	1	1	1	0	1	1	1	1
2452HH	BOK	1	1	0	1	1	1	1	0	1	1	1	1	1	1	1	1	1	1	1	1	1	1	1	1	0	1	1	1	1	1	1	1	1	1	1	1	1	1	1	1	1	1	1	1	1	1	0	1	1	1	1
Ge_alt_VG	BOK	1	1	1	1	1	1	1	0	1	1	1	1	1	1	1	1	1	1	1	1	1	1	1	1	0	1	1	1	1	1	1	1	1	1	1	1	1	1	1	1	1	1	1	1	1	1	0	1	1	1	1
SSK_alt_VG	BOK	1	1	1	1	1	1	1	0	1	1	1	1	1	1	1	1	1	1	1	1	1	1	1	1	1	1	1	0	1	1	1	1	0	1	1	1	1	1	1	1	1	1	1	1	1	1	0	1	1	1	1
SSK_alt_VG	BOK	1	1	1	1	1	1	1	0	1	1	1	1	1	1	1	1	1	1	1	1	1	1	1	1	1	1	1	0	1	1	1	1	0	1	1	1	1	1	1	1	1	1	1	1	1	1	0	1	1	1	1
N.d.M. 2015	INT	1	1	0	1	1	1	1	0	1	1	1	1	1	1	1	1	1	1	1	1	1	1	1	1	1	1	1	1	1	1	1	1	0	1	1	1	1	1	1	1	1	1	1	1	1	1	0	1	1	1	1
Noble 2013	INT	1	1	1	1	1	1	1	0	1	1	1	1	1	1	1	1	1	1	1	1	1	1	1	1	1	1	1	0	1	1	1	1	0	1	1	1	1	1	1	1	1	1	1	1	1	1	0	1	1	1	1
N.d.M.2013	INT	1	1	1	1	1	1	1	0	1	1	1	1	1	1	1	1	1	1	1	1	1	1	1	1	0	1	1	0	1	1	1	1	1	1	1	1	1	1	1	1	1	1	1	1	1	1	0	1	1	1	1
Noble 2015	INT	1	1	1	1	1	1	1	0	1	1	1	1	1	1	1	1	1	1	1	1	1	1	1	1	0	1	1	0	1	1	1	1	1	1	1	1	1	1	1	1	1	1	1	1	1	1	0	1	1	1	1
B04	LEIT	1	1	1	1	1	1	1	1	1	1	1	1	1	1	1	1	1	1	1	1	1	1	1	1	0	1	1	0	1	1	1	1	0	1	1	1	1	1	1	1	1	1	1	1	1	1	0	1	1	1	1
B04	LEIT	1	1	1	1	1	1	1	0	1	1	1	1	1	1	1	1	1	1	1	1	1	1	1	1	0	1	1	1	1	1	1	1	0	1	1	1	1	1	1	1	1	1	1	1	1	1	0	1	1	1	1
D10	LEIT	1	1	1	1	1	1	1	0	1	1	1	1	1	1	1	1	1	1	1	1	1	1	1	1	1	1	1	0	1	1	1	1	0	1	1	1	1	1	1	1	1	1	1	1	1	1	0	1	1	1	1
D10	LEIT	1	1	1	1	1	1	1	0	1	1	1	1	1	1	1	1	1	1	1	1	1	1	1	1	1	1	1	0	1	1	1	1	0	1	1	1	1	1	1	1	1	1	1	1	1	1	0	1	1	1	1
P09	LEIT	1	1	1	1	1	1	1	0	1	1	1	1	1	1	1	1	1	1	1	1	1	1	1	1	1	1	1	1	1	1	1	1	0	1	1	1	1	1	1	1	1	1	1	1	1	1	0	1	1	1	1
P09	LEIT	1	1	1	1	1	1	1	0	1	1	1	1	1	1	1	1	1	1	1	1	1	1	1	1	1	1	1	1	1	1	1	1	0	1	1	1	1	1	1	1	1	1	1	1	1	1	0	1	1	1	1
P10	LEIT	1	1	1	1	1	1	1	0	1	1	1	1	1	1	1	1	1	1	1	1	1	1	1	1	1	1	1	0	1	1	1	1	0	1	1	1	1	1	1	1	1	1	1	1	1	1	0	1	1	1	1
P10	LEIT	1	1	1	1	1	1	1	0	1	1	1	1	1	1	1	1	1	1	1	1	1	1	1	1	1	1	1	1	1	1	1	1	0	1	1	1	1	1	1	1	1	1	1	1	1	1	0	1	1	1	1
P12	LEIT	1	1	1	1	1	1	1	0	1	1	1	1	1	1	1	1	1	1	1	1	1	1	1	1	1	1	1	1	1	1	1	1	0	1	1	1	1	1	1	1	1	1	1	1	1	1	0	1	1	1	1
S20	SCH	1	1	1	1	1	1	1	0	1	1	1	1	1	1	1	1	1	1	1	1	1	1	1	1	1	1	1	1	1	0	1	1	0	1	1	1	1	1	1	1	1	1	1	1	1	1	0	1	1	1	1
S26	SCH	1	1	1	1	1	1	1	0	1	1	1	1	1	1	1	1	1	1	1	1	1	1	1	1	1	1	1	-1	1	1	1	1	0	1	1	1	1	1	1	1	1	1	1	1	1	1	1	1	1	1	1
S26	SCH	1	1	1	1	1	1	1	0	1	1	1	1	1	1	1	1	1	1	1	1	1	1	1	1	1	1	1	0	1	1	1	1	0	1	1	1	1	1	1	1	1	1	1	1	1	1	0	1	1	1	1
SSKB	SCH	1	1	1	1	1	1	1	0	1	1	1	1	1	1	1	1	1	1	1	1	1	1	1	1	1	1	1	1	1	1	1	1	0	1	1	1	1	1	1	1	1	1	1	1	1	1	0	1	1	1	1
SSKB	SCH	1	1	1	1	1	1	1	0	1	1	1	1	1	1	1	1	1	1	1	1	1	1	1	1	1	1	1	0	1	1	1	1	0	1	1	1	1	1	1	1	1	1	1	1	1	1	0	1	1	1	1
K02	SCH	1	1	0	1	1	1	1	0	1	1	1	1	1	1	1	1	1	1	1	1	1	1	1	1	0	1	1	0	1	1	1	1	1	1	1	1	1	1	1	1	1	1	1	1	1	1	0	1	1	1	1
K02	SCH	1	1	0	1	1	1	1	0	1	1	1	1	1	1	1	1	1	1	1	1	1	1	1	1	0	1	1	1	1	1	1	1	1	1	1	1	1	1	1	1	1	1	1	1	1	1	0	1	1	1	1
K02	SCH	1	1	0	1	1	1	1	0	1	1	1	1	1	1	1	1	1	1	1	1	1	1	1	1	0	1	1	1	1	1	1	1	1	1	1	1	1	1	1	1	1	1	1	1	1	1	0	1	1	1	1

Supplement 2: for manuscrip

Sample ID	Sample Set	E-ATA-M16-0183	E-ATA-M16-0177	E-ATA-M16-0172	E-ATA-M16-0167	E-ATA-M16-0134	E-ATA-M16-0123	E-ATA-M16-0108	E-ATA-M19-0349	E-ATA-M19-0288	E-ATA-M19-0266	E-ATA-M19-0248	E-ATA-M19-0220	E-ATA-M19-0210	E-ATA-M19-0201	E-ATA-M19-0191	E-ATA-M19-0170	E-ATA-M19-0150	E-ATA-M19-0125	E-ATA-M19-0117
2422SSK	BOK	1	1	1	0	1	1	1	1	1	1	1	1	0	1	1	1	1	1	1
2425T1	BOK	1	1	1	0	1	1	1	1	1	1	1	1	0	1	1	1	1	1	1
2426T1	BOK	1	1	1	0	1	1	1	1	1	1	1	1	0	1	1	1	1	1	1
2428Ge	BOK	1	1	1	0	1	1	1	1	1	1	1	1	0	1	1	1	1	1	1
2431MRK	BOK	1	1	1	0	1	1	1	1	1	1	1	1	0	1	1	1	1	1	1
2431MRK	BOK	1	1	1	0	1	1	1	1	1	1	1	1	0	1	1	1	1	1	1
2431MRK	BOK	1	1	1	0	1	1	1	1	1	1	1	1	0	1	1	1	1	1	1
2451F12_1	BOK	1	1	1	0	1	1	1	1	1	1	1	1	1	1	1	1	1	1	1
2451F12_1	BOK	1	1	1	0	1	1	1	1	1	1	1	1	1	1	1	0	1	1	1
2452HH	BOK	1	1	1	0	1	1	1	1	1	1	1	1	0	1	1	1	1	1	1
2452HH	BOK	1	1	1	0	1	1	1	1	1	1	1	1	0	1	1	1	1	1	1
2452HH	BOK	1	1	1	0	1	1	1	1	1	1	1	1	0	1	1	1	1	1	1
Ge_alt_VG	BOK	1	1	1	0	1	1	1	1	1	1	1	1	0	1	1	1	1	1	1
SSK_alt_VG	BOK	1	1	1	0	1	1	1	1	1	1	1	1	0	1	1	1	1	1	1
SSK_alt_VG	BOK	1	1	1	0	1	1	1	1	1	1	1	1	0	1	1	1	1	1	1
N.d.M. 2015	INT	1	1	1	0	1	1	1	1	1	1	1	1	0	1	1	1	1	1	1
Noble 2013	INT	1	1	1	0	1	1	0	1	1	1	1	1	0	1	1	1	1	1	1
N.d.M.2013	INT	1	1	1	0	1	1	1	1	1	1	1	1	0	1	1	1	1	1	1
Noble 2015	INT	1	1	1	0	1	1	1	1	1	1	1	1	0	1	1	1	1	1	1
B04	LEIT	1	1	1	0	1	1	1	1	1	1	1	1	0	1	1	1	1	1	1
B04	LEIT	1	1	1	0	1	1	1	1	1	1	1	1	0	1	1	1	1	1	1
D10	LEIT	1	1	1	0	1	1	1	1	1	1	1	1	0	1	1	1	1	1	1
D10	LEIT	1	1	1	0	1	1	1	1	1	1	1	1	0	1	1	1	1	1	1
P09	LEIT	1	1	1	0	1	1	0	1	1	1	1	1	0	1	1	1	1	1	1
P09	LEIT	1	1	1	0	1	1	0	1	1	1	1	1	0	1	1	1	1	1	1
P10	LEIT	1	1	1	0	1	1	1	1	1	1	1	1	0	1	1	1	1	1	1
P10	LEIT	1	1	1	0	1	1	1	1	1	1	1	1	0	1	1	1	1	1	1
P12	LEIT	1	1	1	0	1	1	1	1	1	1	1	1	0	1	1	1	1	1	1
S20	SCH	1	1	1	0	1	1	1	1	1	1	1	1	0	1	1	1	1	1	1
S26	SCH	1	1	1	1	1	1	1	1	1	1	1	1	0	1	1	1	1	1	1
S26	SCH	1	1	1	0	1	1	1	1	1	1	1	1	0	0	1	1	1	1	1
SSKB	SCH	1	1	1	0	1	1	1	1	1	1	1	1	0	1	1	1	1	1	1
SSKB	SCH	1	1	1	0	1	1	1	1	1	1	1	1	0	1	1	1	1	1	1
K02	SCH	1	1	1	0	1	1	1	1	1	1	1	1	0	1	1	1	1	1	1
K02	SCH	1	1	1	0	1	1	1	1	1	1	1	1	0	1	1	1	1	1	1
K02	SCH	1	1	1	0	1	1	1	1	1	1	1	1	0	1	1	1	1	1	1
